# Late Pleistocene speciation of three closely related tree peonies endemic to the Qinling–Daba Mountains, a major glacial refugium in Central China

**DOI:** 10.1002/ece3.5284

**Published:** 2019-06-17

**Authors:** Xing‐Xing Xu, Fang‐Yun Cheng, Li‐Ping Peng, Yan‐Qiang Sun, Xian‐Ge Hu, San‐Yuan Li, Hong‐Li Xian, Kai‐Hua Jia, Richard J. Abbott, Jian‐Feng Mao

**Affiliations:** ^1^ Peony International Research Institute, National Flower Engineering Research Centre, Key Laboratory for the Genetics and Breeding of Forest Trees and Ornamental Plants, College of Landscape Architecture Beijing Forestry University Beijing China; ^2^ Beijing Advanced Innovation Center for Tree Breeding by Molecular Design, National Engineering Laboratory for Tree Breeding, Key Laboratory of Genetics and Breeding in Forest Trees and Ornamental Plants, Ministry of Education, College of Biological Sciences and Technology Beijing Forestry University Beijing China; ^3^ Forestry Department of Shaanxi Province Xi'an Shaanxi China; ^4^ School of Biology, Mitchell Building University of St Andrews St Andrews Fife UK

**Keywords:** ecological niche modeling, genetic divergence, multiple refugia, niche divergence, phylogeography, speciation, tree peony

## Abstract

Determining the factors promoting speciation is a major task in ecological and evolutionary research and can be aided by phylogeographic analysis. The Qinling–Daba Mountains (QDM) located in central China form an important geographic barrier between southern subtropical and northern temperate regions, and exhibit complex topography, climatic, and ecological diversity. Surprisingly, few phylogeographic analyses and studies of plant speciation in this region have been conducted. To address this issue, we investigated the genetic divergence and evolutionary histories of three closely related tree peony species (*Paeonia qiui*, *P. jishanensis*, and *P. rockii*) endemic to the QDM. Forty populations of the three tree peony species were genotyped using 22 nuclear simple sequence repeat markers (nSSRs) and three chloroplast DNA sequences to assess genetic structure and phylogenetic relationships, supplemented by morphological characterization and ecological niche modeling (ENM). Morphological and molecular genetic analyses showed the three species to be clearly differentiated from each other. In addition, coalescent analyses using DIYABC conducted on nSSR variation indicated that the species diverged from each other in the late Pleistocene, while ecological niche modeling (ENM) suggested they occupied a larger area during the Last Glacial Maximum (LGM) than at present. The combined genetic evidence from nuclear and chloroplast DNA and the results of ENM indicate that each species persisted through the late Pleistocene in multiple refugia in the Qinling, Daba, and Taihang Mountains with divergence favored by restricted gene flow caused by geographic isolation, ecological divergence, and limited pollen and seed dispersal. Our study contributes to a growing understanding of the origin and population structure of tree peonies and provides insights into the high level of plant endemism present in the Qinling–Daba Mountains of Central China.

## 1 INTRODUCTION

It is widely known that climatic fluctuations during the Pleistocene strongly impacted the distribution and genetic structure of plant and animal species in both Northern and Southern Hemispheres (Hewitt, 2004; Lu, Heckel, Liang, & Zhang, 2013; Song et al., 2009). Although China experienced less severe glaciations during this period relative to Europe and North America (López‐Pujol, Zhang, Sun, Ying, & Ge, 2011; Qian & Ricklefs, 1999, 2000; Rost, 2000), climatic oscillations nonetheless affected the genetic diversity and evolution of many extant species in the region (Feng, Zheng, & Gong, 2016; Song et al., 2009; Zhou et al., 2010). Recent phylogeographic surveys in China indicate that during Pleistocene glaciations montane species might frequently have persisted in topographically complex regions that served as refugia, rather than being restricted only to lower latitudes (Li et al., 2009; Liu et al., 2013; Qiu, Fu, & Comes, 2011; Song et al., 2009; Zhou et al., 2010). As one of the major areas of endemism in China (López‐Pujol et al., 2011), central China is characterized by a complex topography with altitudes ranging from 100 to 3,767 m. The relatively stable habitats within this region have enabled endemics to persist and also provided conditions for new species to form throughout Quaternary oscillations (López‐Pujol et al., 2011; Ying, 1994; Ying & Zhang, 1993). However, in contrast to numerous phylogeographic surveys of plants from the Qinghai‐Tibet Plateau (Liu et al., 2013; Luo et al., 2016; Zhang et al., 2018) and subtropical China (Fan et al., 2016; Wang et al., 2015) where the effects of climatic oscillations on genetic diversity have been well studied, relatively few phylogeographic analyses of plants in central China (Shao & Xiang, 2015; Zhou et al., 2010) have been conducted, and consequently, little is currently known about mechanisms responsible for the high level of plant endemism found there.

The Qinling–Daba Mountain range, located in central China, extends east‐to‐west for nearly 2,500 km (Dong et al., 2011; Ying, 1994; Yuan, Cheng, & Zhou, 2012), forming an important geographical barrier between China's northern temperate and southern subtropical regions, and representing a major watershed between the Yellow River and Yangtze River (Ma & He, 1988; Rost, 1994, 2000; Xia, 1990). During the Quaternary, the Qinling–Daba Mountains were glacial refugia for such iconic fauna as the giant panda (*Ailuropoda melanoleuca*) and golden takin (*Budorcas taxicolor bedfordi*; Axelrod, Shehbaz, & Raven, 1998; Ying, 1994), and are also thought to have served as refugia for more than 1,620 endemic Chinese plant species (Dong et al., 2011; Ying, 1994). For this reason and because of their geological, climatic, and ecological diversity, the Qinling–Daba Mountains provide an excellent setting for studying factors underlying divergence and speciation during the Pleistocene. Surprisingly, however, relatively few phylogeographic studies of plants (Li, Wan, Guo, Abbott, & Rao, 2014; Shao & Xiang, 2015; Yuan et al., 2012) compared to animals (Fang et al., 2015; Huang et al., 2017; Wang, Jiang, Xie, & Li, 2012, 2013; Yan, Wang, Chang, Xiang, & Zhou, 2010) have been conducted in this region.

**Figure 1:**
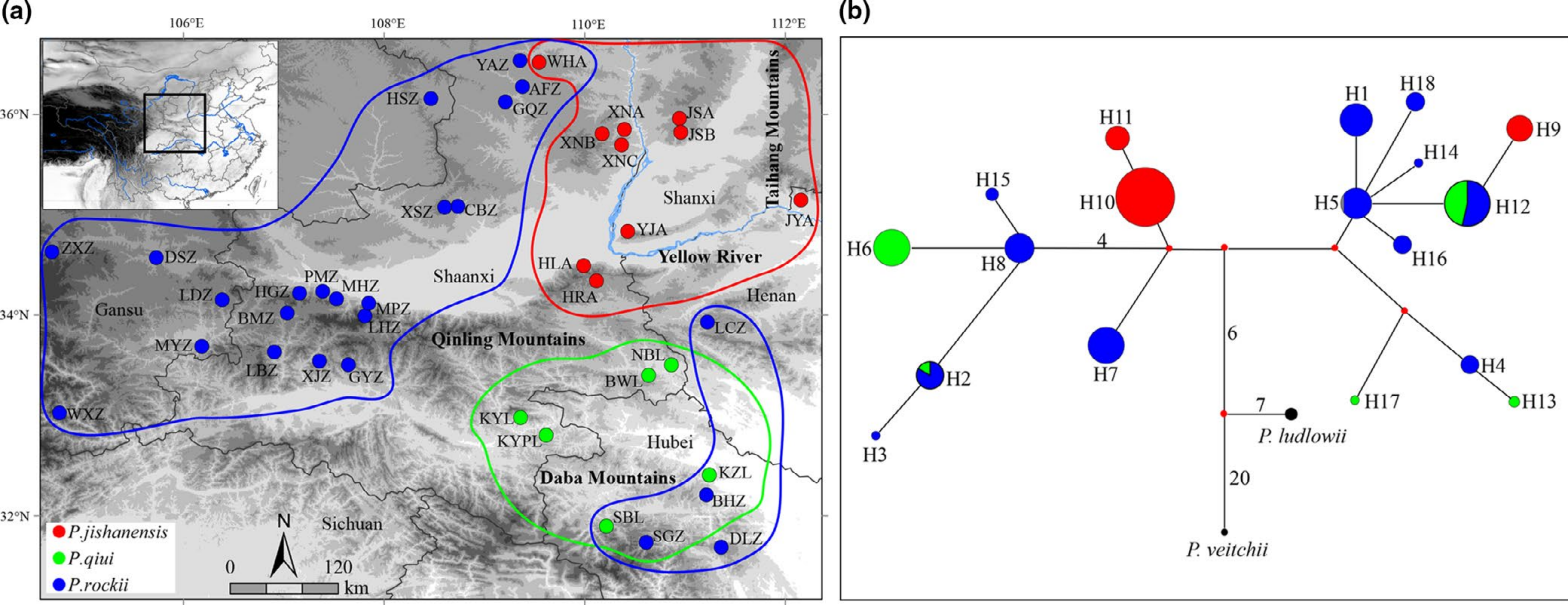
(a) Locations of sampled populations of *P. jishanensis* (red circles)*, P. rockii* (blue circles), and *P. qiui* (green circles). Population codes and locations are presented in Table 1; (b) median‐joining network of chloroplast DNA haplotypes recovered from *P. jishanensis* (red circles), *P. rockii* (blue circles), *P. qiui* (green circles), *P. ludlowii* (black, out‐group), and *P. veitchii* (black, out‐group). Circle size indicates haplotype frequency

Tree peonies, which belong to section *Moutan* of the genus *Paeonia* (Hong & Pan, 2005; Stern, 1946), are distributed in central and northwestern China and include eight wild species (Hong, Pan, & Turland, 2001). Among them, four endemic species, *Paeonia jishanensis* T. Hong & W. Z. Zhao*, Paeonia rockii* (S. G. Haw & Lauener), T. Hong & J. J. Li, *Paeonia qiui* Y. L. Pei & D. Y. Hong, and *Paeonia ostii* T. Hong & J. X. Zhang, are mainly found in the Qinling–Daba Mountains (Hong, 2010; Li, 2011), although *P. ostii* is listed as “critically Endangered” in the Threatened Species List of China's Higher Plants (Qin et al., 2017) with extremely small populations owing to human overexploitation and domestication (Hong & Pan, 1999). Of the remaining species, *Paeonia rockii* is widely distributed throughout a large part of the Qinling–Daba Mountains and extends northwards in China at altitudes between 1,100 and 2,800 m, while *Paeonia jishanensis* and *P. qiui* have more restricted distributions, being confined to the northern and southern Qinling–Daba Mountains (Figure 1a), respectively, at altitudes between 750 and 1,700 m (Hong et al., 2001; Xu, Cheng, Xian, & Peng, 2016).

The three tree peony species are closely related showing minor morphological differences (Hong et al., 2001). *Paeonia qiui* has leaves with nine leaflets that usually lack obvious purple‐red color above and is very similar to *P. jishanensis*, making their separate identification extremely difficult (Xu, Cheng, Peng, & Xian, 2017). Recent molecular phylogenetic analysis has confirmed a close relationship among the three species which are assigned to subsection *Vaginatae* (Zhang, Wang, Xia, & Zhou, 2009; Zhao, Zhou, Lin, Pan, & Li, 2008) with *P. qiui* and *P. jishanensis* considered as the most recently diverged sister species pair (Zhang et al., 2009; Zhao et al., 2008; Zhou et al., 2014). In phylogenetic trees produced from chloroplast DNA sequences, placement of *P. qiui*/*P. jishanensis* and *P. rockii* indicates that introgression may have occurred between them (Zhou et al., 2014). A recent coalescent analysis using approximate Bayesian computation (ABC) of nuclear simple sequence repeat (nSSR) variation indicated that *P. jishanensis* and *P. rockii* diverged from their common ancestor ca. 140K–220K years ago (Yuan, Cornille, Giraud, Cheng, & Hu, 2014). *Paeonia qiui* was not included in this analysis and information on its phylogeography is therefore lacking, while that for *P. jishanensis* and *P. rockii* is limited. The distributions of all three tree peony species have recently been decreasing based on our own field observation (Cheng, 2007; Xu et al., 2017; Yuan et al., 2012). Consequently, they have been listed as rare and endangered species in the Red Book of Chinese Plant Species (Fu, 1992). In addition, *P. qiui* and *P. rockii* have been classified as “endangered” in the Threatened Species List of China's Higher Plants, and *P. jishanensis* is listed as “vulnerable” (Qin et al., 2017). Hence, developing conservation programs for them has become urgent.

Here, we report an investigation of morphological divergence and evolutionary history of the three peony species, *P. qiui, P. jishanensis,* and *P. rockii*, based on nSSR and chloroplast DNA sequence variation. In addition, we examine niche divergence between the species using ecological niche modeling (ENM) and compare spatial distributions at present with those at the Last Glacial Maximum (LGM). Our research aimed to (a) investigate patterns of interspecific and intraspecific genetic differentiation among the three species; (b) estimate times of divergence; (c) test modes of origin and infer the demographic history of each species; and (d) consider effective conservation and management strategies separately for each of the three peony species in Central China.

## 2 MATERIALS AND METHODS

### 2.1 Sampling and sequencing

Leaves were sampled from 587 individuals across 40 populations, including 255 individuals from 10 populations of *P. jishanensis*, 118 individuals from 6 populations of *P. qiui*, and 214 individuals from 24 populations of *P. rockii*. Sampling covered the Qinling–Daba Mountains and adjacent areas (Table 1, Figure 1a). Total genomic DNA was extracted from silica gel‐dried leaf tissue using a DNeasy Plant Mini kit (Tiangen Biotech). Individuals were genotyped using 22 EST‐SSR markers (Table S1) distributed over five linkage groups according to the tree peony high‐density genetic linkage map (Cai, 2015; Peng et al., 2017). Details of nuclear microsatellite genotyping are described in Wu, Cai, Cheng, Cui, and Zhou (2014). In addition, three chloroplast (cp) DNA intergenic spacers (*pet*B‐*pet*D, *acc*D‐*psa*I, and *psb*E‐*pet*L) were sequenced across a subsample of 296 individuals (Table 1) using the methods described in Grivet, Heinze, Vendramin, and Petit (2001) and Suo et al. (2012). All cpDNA sequences have been deposited in GenBank under accession numbers of KY200296–KY200338.

**Table 1:** Geographic locations and sample sizes of 40 *P. jishanensis*, *P. qiui,* and *P. rockii* populations used in this study

Species	Population code	Location	*N*_nuc_	*N*_cp_	Latitude(°N)	Longitude(°E)	Altitude (m)
*P. jishanensis*	WHA	Mt.Wanhua, Shaanxi	36	8	36.520	109.342	1,066
*P. jishanensis*	XNA	Mt.Nan, Shaanxi	16	7	35.941	110.396	752
*P. jishanensis*	XNB	Mt.Caizi, Shaanxi	39	10	35.930	110.404	950
*P. jishanensis*	XNC	Mt.Mangtou, Shaanxi	20	7	35.992	110.392	1,066–1,366
*P. jishanensis*	JSA	Mapaoquan, Shanxi	31	10	35.634	110.966	1,200
*P. jishanensis*	JSB	Majiagou, Shanxi	35	13	35. 616	110.950	1,250
*P. jishanensis*	JYA	Heilonggou, Henan	25	15	35.261	112.078	1,054
*P. jishanensis*	HRA	Mt.Hua, Shaanxi	6	6	34.435	110.069	1,300
*P. jishanensis*	HLA	Luofu, Shaanxi	21	10	34.490	109.988	895
*P. jishanensis*	YJA	Shuiyukou, Shanxi	26	13	34.834	110.431	1,000–1,650
*P. qiui*	BWL	Shangnan, Shaanxi	26	13	33.399	110.637	944–1,050
*P. qiui*	NBL	Shangnan, Shaanxi	29	13	33.673	111.041	770–804
*P. qiui*	KYPL	Xunyang, Shaanxi	16	5	32.980	109.376	1,471
*P. qiui*	KYL	Xunyang, Shaanxi	36	14	32.980	109.358	1,525
*P. qiui*	SBL	Shennongjia, Hubei	5	3	31.736	110.600	1,561
*P. qiui*	KZL	Baokang, Hubei	6	5	32.449	111.455	500
*P. rockii*	DLZ	Dashui, Hubei	29	5	31.687	111.3477	1,622
*P. rockii*	BHZ	Hengchong, Hubei	10	3	32.209	111.202	1,742
*P. rockii*	SGZ	Songbai, Hubei	10	8	31.736	110.600	1,561
*P. rockii*	LCZ	Luanchuan, Henan	4	4	33.931	111.211	1,100–1,200
*P. rockii*	MYZ	Huixian, Gansu	9	6	33.690	106.170	1,200–1,373
*P. rockii*	WXZ	Wenxian, Gansu	10	8	33. 027	104.752	1,675
*P. rockii*	LDZ	Liangdang, Gansu	10	8	34.151	106.520	1,505–1,670
*P. rockii*	LBZ	Liuba, Shaanxi	11	3	33.833	107.094	1,200
*P. rockii*	MPZ	Yangxian, Shaanxi	10	8	34.005075	107.315	1,341
*P. rockii*	GYZ	Erlangba, Shaanxi	5	3	33.727	107.419	1,055
*P. rockii*	XJZ	Xiangjiagou, Shaanxi	6	3	33.724	107.385	1,400–1,600
*P. rockii*	ZXZ	Zhangxian, Gansu	10	10	34.631	104.676	1881–1942
*P. rockii*	DSZ	Tianshui, Gansu	6	4	34.571	105.717	1,393–1,553
*P. rockii*	HSZ	Heshui, Gansu	10	10	36.007	108.653	1,312–1,362
*P. rockii*	YAZ	Wanhuashan, Shaanxi	3	3	36.537	109.342	1,215
*P. rockii*	CBZ	Yaoxian, Shaanxi	10	8	35.082	108.725	1,134–1,252
*P. rockii*	GQZ	Ganquan, Shaanxi	10	9	36.277	109.365	1,370–1,444
*P. rockii*	AFZ	Fuxian, Shaanxi	10	9	36.128	109.198	1,237
*P. rockii*	BMZ	Mt. Maer, Shaanxi	7	4	34.053	107.646	1,471–1,709
*P. rockii*	LHZ	Mt. Lianhuafeng, Shaanxi	4	4	34.053	107.900	1,000
*P. rockii*	PMZ	Mt. Pomo, Shaanxi	5	3	34.053	107.890	900
*P. rockii*	HGZ	Heihuguan, Shaanxi	6	5	34.096	107.679	900
*P. rockii*	MHZ	Haoping, Shaanxi	9	8	34.093	107.719	1,245–1,646
*P. rockii*	XSZ	Xunyi, Shaanxi	10	8	35.073	108.593	1,728
Total			587	296			

### 2.2 Morphological differences

Six floral traits (color of petal, carpel, filament, stigma and flare on the petal base, and number of carpels) and seven vegetative characters (leaf color, type of compound leaves, stolon, lobed leaflet, shape of tip leaflet, number of leaflets, and plant height) were selected for recording (Table S2A). Each trait was recorded on 39 individuals across all three species (Table S2B). Differences for each trait among species were evaluated using the Kruskal–Wallis multiple‐range test in Agricolae (De, 2014) and vegan (Oksanen et al., 2013). In addition, principal component analysis (PCA) was performed to examine multivariate differences between species. All statistical analyses were performed in *R* (R Core Team, 2015).

### 2.3 Chloroplast DNA sequence analysis

Sequences were edited and aligned using MEGA 6.0 (Tamura, Stecher, Peterson, Filipski, & Kumar, 2013). Haplotype diversity (*H*_d_) and nucleotide diversity (π) were estimated using DNASP 5.0 (Librado & Rozas, 2009). The three cpDNA regions were combined after a homogeneity test in PAUP* 4.0b10 (Swofford, 2002) showed no significant phylogenetic heterogeneity between them (*p* = 0.1, >0.05). Haplotype networks were constructed using NETWORK 4.5.1 (Bandelt, Forster, & Röhl, 1999) with gaps (indels) coded as substitutions (A or T). Analyses of molecular variance (AMOVA) were carried out using ARLEQUIN 3.5 (Excoffier & Lischer, 2010) to examine the distribution of variation within and between species. Phylogeographical structure was estimated by testing whether *N*_ST _was significantly larger than *G*_ST_ using PERMUT 1.0 with 10,000 permutations (Pons & Petit, 1996).

### 2.4 Phylogenetic analysis and estimation of divergence time

Phylogenetic relationships and divergence times between cpDNA haplotype lineages were estimated using Bayesian inference methods implemented in BEAST 1.8.0 (Drummond, Suchard, Xie, & Rambaut, 2012) with *Paeonia ludlowii* and *P. veitchii* used as out‐groups. The HKY substitution model selected by JMODELTEST 2.1.7 (Darriba, Taboada, Doallo, & Posada, 2012) was used and a Yule process specified tree prior. With no peony fossils available, a mutation rate of 1.01 × 10^−9^ per site per year estimated for seed plants (Graur & Li, 2000) was applied to calculate divergence times. Parameters were sampled every 1,000 steps for 10^7 ^MCMC steps, with the first 10% of samples discarded as burn‐in. Reliability was evaluated by testing for effective sample size (ESS) using TRACER 1.5 (Rambaut & Drummond, 2009).

Tajima's *D* (Tajima, 1989) and Fu's *Fs* (Fu, 1997) tests implemented in ARLEQUIN 3.5 were used to determine that all mutations were selectively neutral and to evaluate the hypothesis of demographic expansion. Demographic processes were examined using mismatch distribution analysis in ARLEQUIN 3.5. Multimodal mismatch distributions of pairwise differences between haplotypes indicate that a population size remained relatively stable over time, whereas a unimodal distribution implies a recent demographic expansion (Fu, 1997; Tajima, 1989). The validity of the estimated demographic model was tested by determining the distribution of the sum of squared deviations (*SSD*) between the observed and expected distributions and Harpending's raggedness index (*H*_Rag_; Harpending, 1994).

### 2.5 Microsatellite data analysis

Microsatellite (nSSR) data were checked for null alleles using MICRO‐CHECKER 2.2.3 (Van Oosterhout, Hutchinson, Wills, & Shipley, 2004). Tests for linkage disequilibrium were performed using GENEPOP 4.2 (Rousset, 2008). Analyses of neutrality based on *F*_ST_ outliers under the assumption of the infinite allele model were implemented in LOSITAN, with 50,000 simulations (Antao, Lopes, Lopes, Beja‐Pereira, & Luikart, 2008; Beaumont & Nichols, 1996). Genetic diversity indices were assessed by calculating observed heterozygosity (*H*_O_), expected heterozygosity (*H*_E_), and the number of private alleles (*A*_P_) using GenAlEx (Peakall & Smouse, 2012). Allelic richness (*A*_R_) and the fixation index (*F*_IS_) were estimated with FSTAT 2.9.3 software (Goudet, 2001).

First, population structure was examined using STRUCTURE 2.3.4 (Pritchard, Stephens, & Donnelly, 2000). For all populations of three peony species, we computed the likelihood for *K* clusters from 1 to 20 with ten replicates per *K*. For each species, ten replications were conducted for each *K* in the range *K* = 1–10. Each run had a burn‐in of 100,000 steps, followed by 200,000 MCMC iterations using the admixture model and assuming correlated allele frequencies. The optimal *K* was evaluated according to the methods of Pritchard et al. (2000) and Evanno, Regnaut, and Goudet (2005). The estimated admixture coefficients across replicated runs were permuted using CLUMPP v.1.1.2 (Jakobsson & Rosenberg, 2007). Graphics were produced using DISTRUCT v.1.1 (Rosenberg, 2004). To detect genetic grouping further, principal component analysis (PCA) was also conducted for all and each species implemented with GenALEx 6.5. An unrooted neighbor‐joining tree was also generated based on the shared‐allele distance using PowerMarker 3.25 (Liu & Muse, 2005) to construct genetic relationships among individuals. To examine the partition of nSSR variation within and between species, hierarchical analysis of molecular variance (AMOVA) with 10,000 permutations was conducted using ARLEQUIN 3.5. We estimated pairwise *F*_ST_ values between populations for all peony species in FSTAT 2.9.3 software (Goudet, 2001). This was followed by an evaluation of isolation by distance (IBD) between populations by testing the correlation between the matrix of pairwise *F*_ST_/(1‐*F*_ST_) and the matrix of geographic distances using GENALEX 6.5 with 999 permutations.

The directional relative migration network implemented in divMigrate (Sundqvist, Keenan, Zackrisson, Prodöhl, & Kleinhans, 2016) was used to estimate potential past gene flow among three peony species.

### 2.6 Demographic model test with ABC

**Figure 2:**
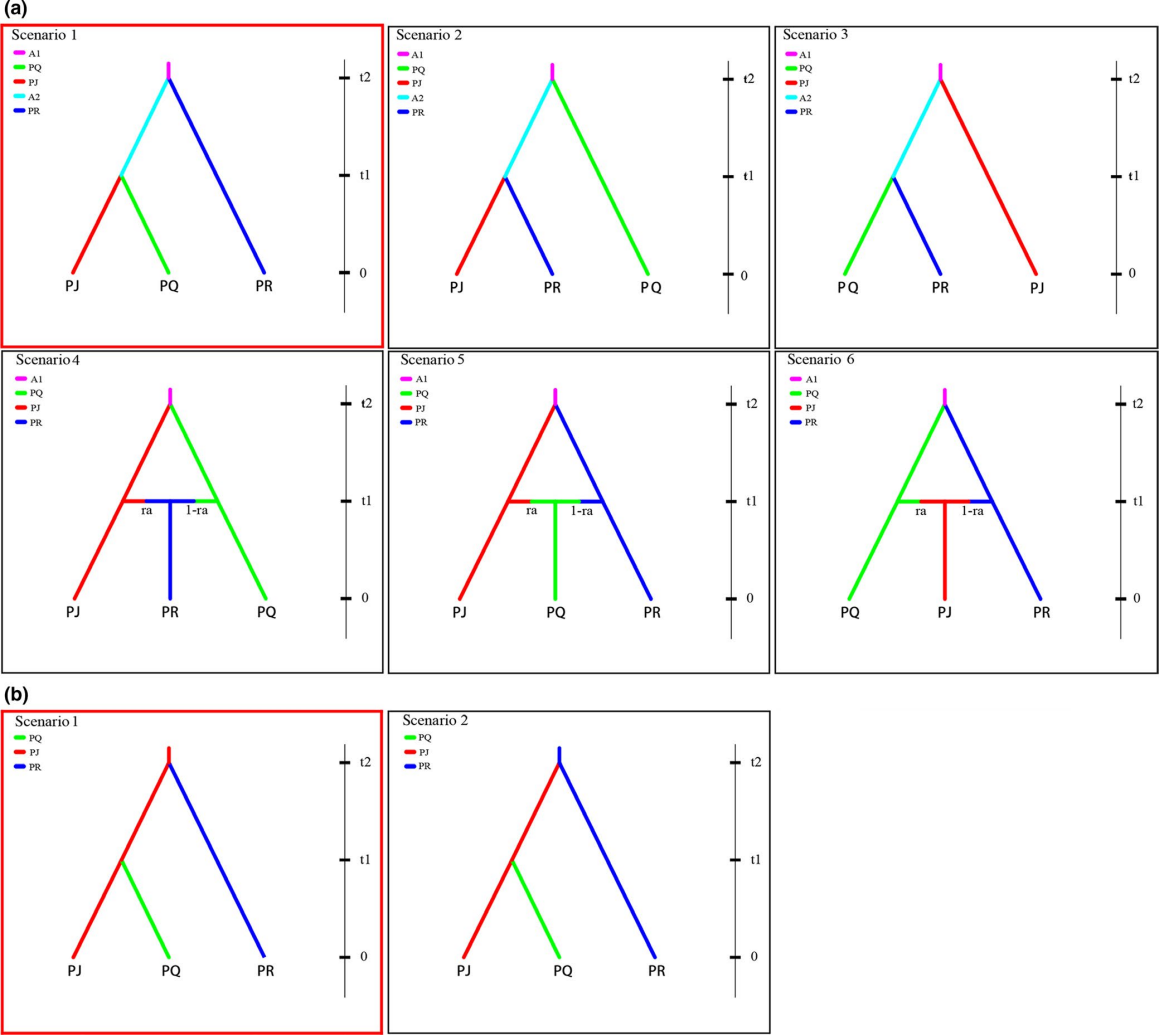
Evolutionary scenarios tested for the origin of *P. jishanensis* (PJ), *P. qiui* (PQ), and *P. rockii* (PR) using approximate Bayesian computation (ABC). In the analysis, current population sizes of the three taxa PJ, PQ, and PR were denoted as NJ, NQ, and NR, respectively, A1 represented the population size of the ancestral population at t2, and A2 represented the population size of ancestral population at t1. The size of an ancestral population was varied in all scenarios. (a) Six scenarios for the origins and relationships among three peony species. (b) Two competing scenarios based on the best‐fit scenario of PQ from PJ. Scenario 1 predicted that PQ split from PJ at time t1, while PR split from PJ at time t2. *P. jishanensis* is the ancestor of *P. rockii*. Scenario 2 predicted that PQ split from PJ at time t1, while PJ split from PR at time t2. In scenario 2, *P. rockii* is the ancestor of *P. jishanensis*

To compare plausible scenarios of divergence and to infer historical parameters of genetic divergence among the three species, we used DIYABC 2.0 (Cornuet et al., 2014) on the microsatellite dataset. Different evolutionary scenarios were constructed, taking account of the results obtained from the morphological divergence and STRUCTURE analyses. In step 1, six possible scenarios among the three species were tested; these differed in the relationships among taxa (Figure 2a). Subsequently, in step 2, two competing scenarios based on the previous best‐fit scenario that *P. qiui* diverged from a common ancestor with *P. jishanensis* were compared (Figure 2b). In each scenario, current population sizes of the three taxa *P. jishanensis, P. qiui, and P. rockii* were denoted as NJ, NQ, and NR, respectively, while A1 represented the population size of the ancestral population at t2, and A2 represented the population size of ancestral population at t1 (Figure 2). Because individuals of tree peony start flowering 5 to 8 years after germination and vegetative reproduction by rhizomes is very common (Sang, Crawford, & Stuessy, 1997), generation time was set at 10 years for all three species in our models.

We assumed a uniform prior distribution for mean mutation rate (*μ*) from 10^−4^ to 10^−3^, which is consistent with rates for woody plants (Petit & Hampe, 2006). Summary statistics used for analysis were mean number of alleles, mean genetic diversity, *F*_ST_, and (dμ)^2^ distance. The posterior probability of the competing scenarios was compared using a polychotomous logistic regression (Fagundes et al., 2007). For the best supported scenario, we estimated distributions of all parameters using 1% of simulated datasets closest to the observed dataset after a logit transformation to parameter values. Confidence in scenario choice was assessed by computing type I error and type II error in the selection of scenarios. Posterior model checking was performed on the selected scenario using 1% of the simulated datasets closest to the observed data (Beaumont, Zhang, & Balding, 2002).

### 2.7 Ecological niche modeling

To predict the geographic distribution of suitable habitat for each species, ecological niche modeling was conducted with MAXENT v. 3.3.3k (Phillips, Anderson, & Schapire, 2006). The occurrence (“occurrence record”) of each species was obtained from field surveys during 2006–2015 and from the Chinese Virtual Herbarium (http://www.cvh.org.cn/). In total, we collected 86 occurrence records: 39 for *P. jishanensis*, 30 for *P. qiui*, and 58 for *P. rockii* (Table S3). The 19 bioclimatic variables for the present time and LGM (c. 21,000 years BP) were downloaded from WorldClim (http://www.worldclim.org/). We examined the correlation between all layers using ENMTOOLS 1.3 (Warren, Glor, & Turelli, 2010). Two variables were regarded to be highly correlated when the correlation coefficient was ≥0.8. Accordingly, only eight variables with correlation coefficients of *r* ≤ 0.8 were used (Table S4): mean diurnal range (Bio2), isothermality (Bio3), temperature seasonality (Bio4), the maximum temperature of the warmest month (Bio5), mean temperature of the driest quarter (Bio9), precipitation of the wettest month (Bio13), precipitation seasonality (Bio15), and precipitation of the coldest quarter (Bio19). The same eight bioclimatic variables were used for the Community Climate System Model (Collins et al., 2010). We ran 20 replicates with 25% of the occurrence points used for model testing and determined logistic probabilities for the output. A presence–absence map was produced using the “maximum training presence threshold.” The area under the curve (AUC) was used to evaluate model performance.

### 2.8 Niche divergence test

To detect niche divergence among species, we performed a niche space‐based multivariate test (Mccormack, Zellmer, & Knowles, 2010) to compare background divergence (*d*_b_) with observed niche divergence (*d*_n_) in the PCA‐reduced axes, with the null hypothesis *d*_b_ = *d*_n_. Niche divergence is supported if *d*_b_ < *d*_n_ and the observed niche divergence itself (*d*_n_) is significant, whereas niche conservatism is supported if *d*_b_ > *d*_n_ (Mao & Wang, 2011).

## 3 RESULTS

### 3.1 Morphological divergence

**Figure 3:**
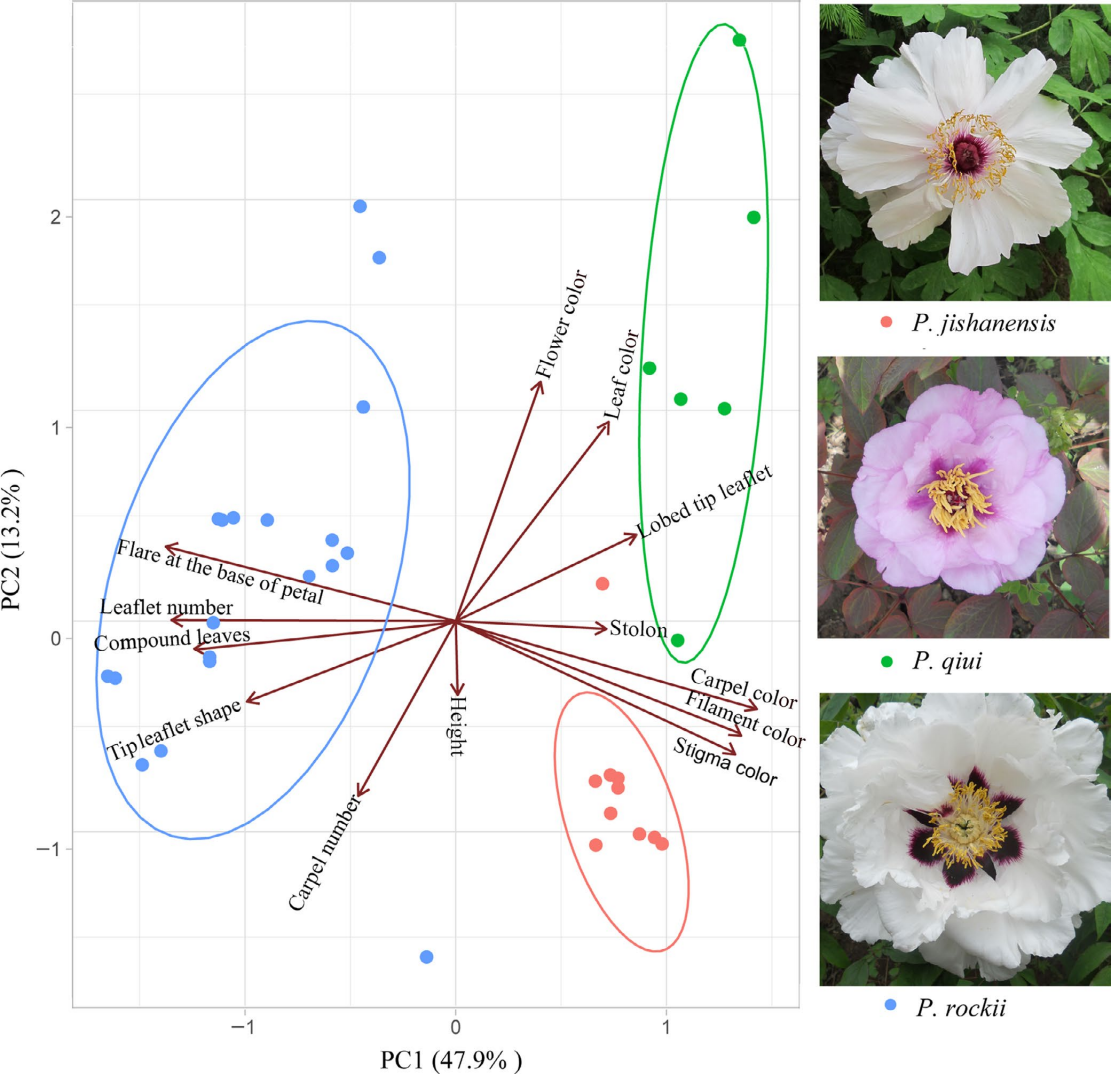
Principal component analysis biplot of scores for PC1 and PC2 for the 39 specimens of three peony species based on an analysis of 13 morphometric traits. Variable are defined in Table S2A. Photographs to the right show the floral differences between *P. jishanensis*, *P. rockii,* and *P. qiui*

ANOVAs and multiple‐range tests (Table 2; Table S5) showed that there were significant differences between species for all floral and vegetative traits recorded except plant height. Greatest divergence in terms of the proportion of total variance due to differences between species was evident for flare of the petal base (95%), carpel color (90%), filament color (90%), stigma color (90%), leaf color (80%), and numbers of carpels (61%; Table 2). However, only for number of leaflets were all three species significantly different from each other according to multiple‐range tests (Table S5). PCA showed that the first two components (PC1 and PC2) accounted for 47.9% and 13.2% of the total variation (Figure 3), and that the 39 individuals were divided into three clearly separated clusters, corresponding to species designations. PC1 distinguished *P. rockii* from *P. jishanensis* and *P. qiui,* while PC2 separated *P. jishanensis* from *P. qiui*. The contributions of each character to each principal component are listed in Table S6, showing that carpel, filament, and stigma color together with flare of petal base, number of leaflets, and type of compound leaves have the highest loadings on PC1, while petal and leaf color together with number of carpels contribute most to the variance explained by PC2.

**Table 2:** ANOVA for 13 morphological characters among *P. jishanensis*, *P. qiui,* and *P. rockii*

Traits	Source	*df*	SS	*F*	Percentage of variance
Flower color of petals	Species	2	3.69	7.72**	0.30
	Residuals	36	8.61		0.70
	Total	38	12.30		
Color of leafs	Species	2	3.52	76.15***	0.81
	Residuals	36	0.83		0.19
	Total	38	4.35		
Color of carpels	Species	2	8.74	166.15***	0.90
	Residuals	36	0.95		0.10
	Total	38	9.69		
Color of filaments	Species	2	8.74	166.15***	0.90
	Residuals	36	0.95		0.10
	Total	38	9.69		
Color of stigma	Species	2	8.74	166.15***	0.90
	Residuals	36	0.95		0.10
	Total	38	9.69		
Type of compound leaves	Species	2	3.26	12.69***	0.41
	Residuals	36	4.63		0.59
	Total	38	7.89		
Flare at the base of petal	Species	2	35.21	359.74***	0.95
	Residuals	36	1.76		0.05
	Total	38	36.97		
Stolon	Species	2	1.22	5.69**	0.24
	Residuals	36	3.85		0.76
	Total	38	5.07		
Lobed or not in tip leaflet	Species	2	0.97	4.26*	0.19
	Residuals	36	4.10		0.81
	Total	38	5.07		
Shape of tip leaflet	Species	2	1.73	6.71**	0.27
	Residuals	36	4.63		0.73
	Total	38	6.36		
Numbers of leaflets	Species	2	1,490.54	28.11***	0.61
	Residuals	36	954.37		0.39
	Total	38	2,444.92		
Numbers of carpels	Species	2	1.26	6.53*	0.27
	Residuals	36	3.5		0.73
	Total	38	4.76		
Height of plant	Species	2	3,394.82	0.85	0.05
	Residuals	36	71,723.95		0.95
	Total	38	75,118.77		

Note

* 0.01 < *p*< 0.05

** 0.001 < *p*< 0.01

*** *p*< 0.001.

### 3.2 Chloroplast DNA variation

A total of 2075 base pairs comprised the concatenated cpDNA sequence surveyed across 296 individuals. Nucleotide substitutions occurred at 37 sites, with one indel present in the *acc*D‐*psa*I region (Table S7). Average diversity was significantly higher in *P. qiui* (*H*_d_ = 0.243; π = 0.002) than in *P. jishanensis* (*H*_d_ = 0.013; π = 0.000006) and *P. rockii* (*H* = 0.110; π = 0.00038; Table 3). A total of 18 cpDNA haplotypes were identified (Figure S1A, B), of which two were shared between *P*. *rockii* and *P. qiui* (H2 and H12), and three, three, and 10 were unique to *P. jishanensis* (H9, H10, and H11), *P. qiui* (H6, H13, and H17) and *P. rockii* (H1, H3, H4, H5, H7, H8, H14, H15, H16, and H18), respectively. The haplotype network grouped the 18 cpDNA haplotypes into two groups and indicated a lack of phylogenetic clustering among populations of the same species. Hierarchical AMOVA of cpDNA variation revealed significant differentiation (*F*_ST_ = 0.929, *p* < 0.0001) with 17.80% of the observed variation partitioned among species, and 75.11% due to variation among populations within species (Table 4). For each species, AMOVA revealed the highest genetic variation partitioned among populations in *P. jishanensis* (99.30%), followed by *P. rockii* (89.31%) and *P. qiui* (89.15%). PERMUT analyses indicated that signatures of phylogeographical structure varied across species. In *P. jishanensis*, *N*_ST_（0.990 ± 0.011）was significantly higher than *G*_ST_ (0.973 ± 0.0216), indicating significant phylogeographical structure. However, there was no significant phylogeographical structure in *P. rockii* and *P. qiui*; in each instance *N*_ST_ (*P. rockii*: 0.872 ± 0.066; *P. qiui*: 0.711 ± 0.171）was not significantly higher than *G*_ST _(0.879 ± 0.051; *P. qiui*: 0.794 ± 0.114).

**Table 3:** Measures of genetic diversity for 40 *P. jishanensis*, *P. qiui,* and *P. rockii* populations based on analyses of 22 nuclear microsatellite loci and three cpDNA sequences

Species	Population										
	Code	Microsatellites							cpDNA		
		*N*_nuc_	*H*_O_	*H*_E_	*A*_R_	*A*_P_	*F*_IS_	*N*cp	Hap	*H*_d_	π × 10^3^
*P. jishanensis*	WHA	36	0.419	0.228	1.232	0	−0.832	8	H10(8)	0.000	0.00
*P. jishanensis*	XNA	16	0.356	0.308	1.286	0	−0.157	7	H10(7)	0.000	0.00
*P. jishanensis*	XNB	39	0.407	0.316	1.366	0	−0.045	10	H10(10)	0.000	0.00
*P. jishanensis*	XNC	20	0.419	0.367	1.345	0	−0.226	7	H10(7)	0.000	0.00
*P. jishanensis*	JSA	31	0.385	0.451	1.353	1	−0.041	10	H10(10)	0.000	0.00
*P. jishanensis*	JSB	35	0.406	0.437	1.396	0	−0.125	13	H10(13)	0.000	0.00
*P. jishanensis*	JYA	25	0.475	0.466	1.327	1	−0.692	15	H10(1), H11(14)	0.133	0.06
*P. jishanensis*	HRA	6	0.389	0.371	1.241	0	−0.770	6	H9(6)	0.000	0.00
*P. jishanensis*	HLA	21	0.325	0.344	1.295	0	−0.200	10	H9(10)	0.000	0.00
*P. jishanensis*	YJA	26	0.476	0.424	1.397	0	−0.027	13	H10(13)	0.000	0.00
Mean			0.406	0.371	1.324	0.2	−0.312			0.473	1.24
*P. qiui*	BWL	26	0.510	0.413	1.422	1	−0.214	13	H6(13)	0.000	0.00
*P. qiui*	NBL	29	0.394	0.387	1.431	2	−0.162	13	H6(13)	0.000	0.00
*P. qiui*	KYPL	16	0.375	0.343	1.513	0	0.105	5	H12(5),	0.000	0.00
*P. qiui*	KYL	36	0.408	0.377	1.387	0	−0.010	14	H12(14)	0.000	0.00
*P. qiui*	SBL	5	0.514	0.557	1.579	5	0.219	3	H2(1), H17(2)	0.677	4.91
*P. qiui*	KZL	6	0.463	0.582	1.609	2	−0.028	5	H2(1), H13(4),	0.400	2.76
Mean			0.444	0.443	1.490	1.7	−0.015			0.634	3.37
*P. rockii*	DLZ	29	0.164	0.215	1.220	2	0.260	5	H2(1),H8(4)	0.400	0.58
*P. rockii*	BHZ	10	0.208	0.439	1.483	0	0.598	3	H2(1),H3(1),H4(1)	1.000	4.83
*P. rockii*	SGZ	10	0.282	0.377	1.398	2	0.321	8	H2(8)	0.000	0.00
*P. rockii*	LCZ	4	0.273	0.178	1.203	0	−0.426	4	H15(4)	0.000	0.00
*P. rockii*	MYZ	9	0.348	0.349	1.370	2	0.060	6	H4(1), H12(5)	0.333	1.13
*P. rockii*	WXZ	10	0.238	0.281	1.300	3	0.225	8	H18(8)	0.000	0.00
*P. rockii*	LDZ	10	0.340	0.352	1.346	0	−0.003	8	H12(8)	0.000	0.00
*P. rockii*	LBZ	11	0.303	0.346	1.362	1	0.109	3	H5(2), H14(1)	0.000	0.00
*P. rockii*	MPZ	10	0.293	0.338	1.382	0	−0.097	8	H5(7), H8(1)	0.250	0.97
*P. rockii*	GYZ	5	0.410	0.424	1.305	1	−0.059	3	H5(3)	0.000	0.00
*P. rockii*	XJZ	6	0.265	0.267	1.312	0	0.118	3	H5(3)	0.000	0.00
*P. rockii*	ZXZ	10	0.249	0.349	1.368	0	0.335	10	H4(1), H12(9)	0.200	0.68
*P. rockii*	DSZ	6	0.407	0.359	1.478	0	−0.047	4	H4(4)	0.000	0.00
*P. rockii*	HSZ	10	0.136	0.163	1.257	0	0.286	10	H1(1), H7(9)	0.200	0.77
*P. rockii*	YAZ	3	0.458	0.469	1.409	1	0.135	3	H8(3)	0.000	0.00
*P. rockii*	CBZ	10	0.245	0.252	1.211	0	−0.079	8	H7(8)	0.000	0.00
*P. rockii*	GQZ	10	0.355	0.356	1.416	0	−0.096	9	H8(9)	0.000	0.00
*P. rockii*	AFZ	10	0.492	0.412	1.407	0	0.026	9	H1(9)	0.000	0.00
*P. rockii*	BMZ	7	0.350	0.452	1.392	1	0.102	4	H5(4)	0.000	0.00
*P. rockii*	LHZ	4	0.311	0.254	1.368	0	0.121	4	H16(4)	0.000	0.00
*P. rockii*	PMZ	5	0.287	0.414	1.326	1	0.267	3	H16(3)	0.000	0.00
*P. rockii*	HGZ	6	0.231	0.330	1.300	0	0.055	5	H1(5)	0.000	0.00
*P. rockii*	MHZ	9	0.345	0.385	1.408	0	0.219	8	H1(7), H5(1)	0.250	0.24
*P. rockii*	XSZ	10	0.203	0.255	1.206	2	0.133	8	H7(8)	0.000	0.00
Mean			0.300	0.334	1.343	0.7	0.098			0.874	2.94

Note

*N*_nuc_ and *N*_cp_, the number of samples analyzed for nuclear microsatellites and cpDNA; *H*_O_, mean observed heterozygosity; *H*_E,_ mean expected heterozygosity; *A*_R,_ allelic richness; *A*_P_, number of private alleles; *F*_IS,_ the fixation index; *H*_d_, haplotype diversity; π, nucleotide diversity.

**Table 4:** Hierarchical analysis of molecular variance (AMOVA) conducted on nSSR and cpDNA datasets

Source of variation	nSSR				cpDNA			
	*df*	SS	*V_c_*	%	*df*	SS	*V_c_*	%
nSSRs								
Among species	2	881.240	0.999	20.66***	2	170.372	0.635	17.80***
Among populations within species	37	1560.967	1.415	29.23***	37	724.999	2.680	75.11***
Within populations	1,134	2,751.005	2.426	50.11	256	64.750	0.253	7.09
*P. jishanensis*								
Among populations	9	873.244	1.861	34.04***	9	131.814	1.49202	99.30***
Within populations	500	1803.551	3.607	65.96	89	0.933	0.01049	0.70
*P. qiui*								
Among populations	5	243.686	1.273	37.13***	5	168.050	3.962***	89.15***
Within populations	230	495.738	2.155	62.87	47	22.667	0.48227	10.85
*P. rockii*								
Among populations	23	386.603	0.8858	42.18***	23	393.697	2.818	89.31***
Within populations	404	490.593	1.2143	57.82	120	40.483	0.337	10.69

Note

*df*, degrees of freedom; SS, sum of squares; *V_c_*, variance components; significance: ^***^ *p* < 0.0001; ^**^ *p* < 0.01.

### 3.3 Divergence time and demographic history based on cpDNA data

**Figure 4:**
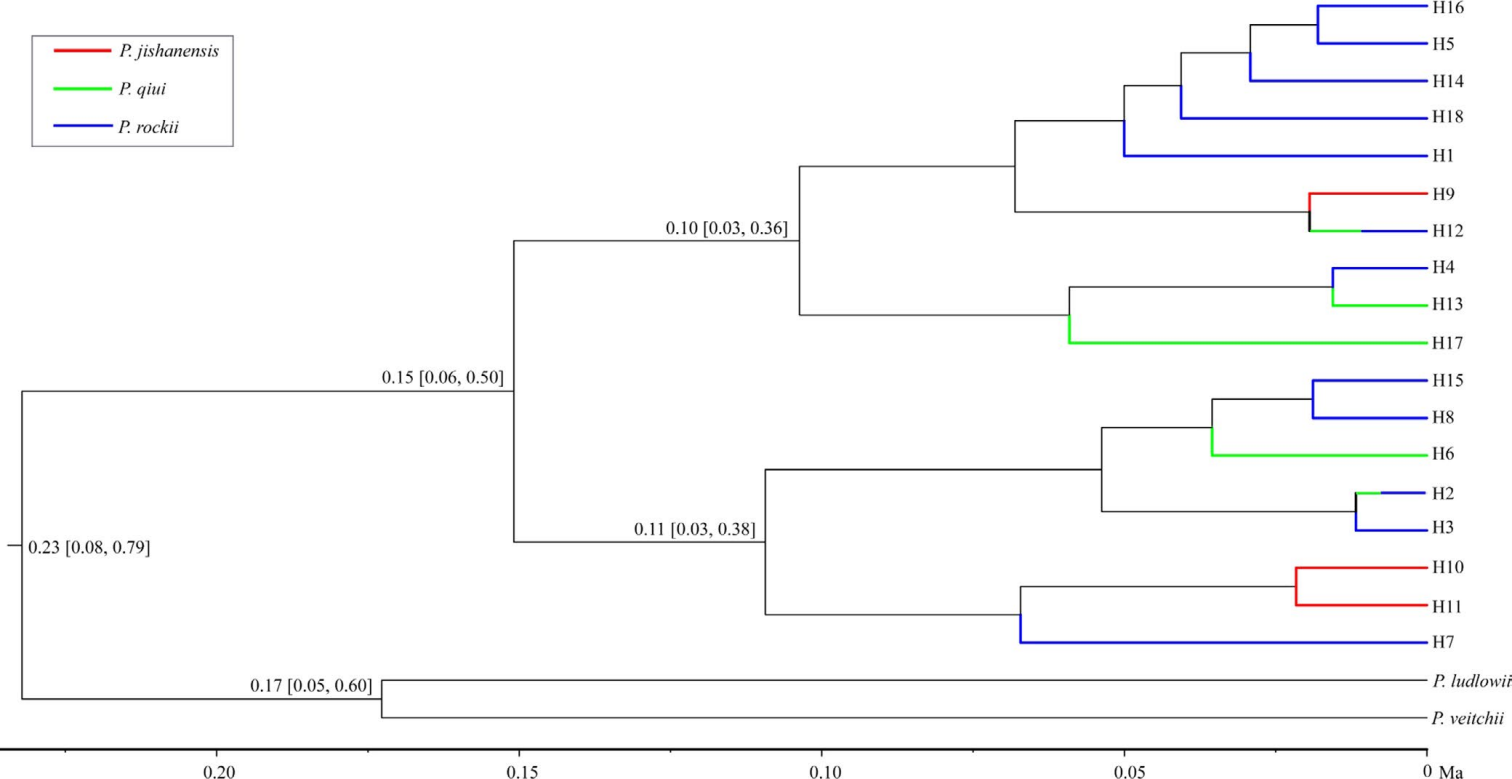
BEAST‐derived trees among the three peony species based on cpDNA haplotypes

Analysis of cpDNA haplotype variation using BEAST detected two clades with relatively high support (posterior probability, *PP* = 0.84; Figure 4). Populations of the same species contained haplotypes that were not always phylogenetically clustered, and the two clades comprised haplotypes found in all three species. Divergence between the two cpDNA clades was dated at 0.15 MYA (95% HPD: 0.06–0.50 MYA).

Tajima's *D* and Fu's *Fs* estimated for each species were non‐significantly positive (Table 5), and the observed mismatch distributions of pairwise nucleotide differences were multimodal (Figure S2). The sum of squares deviations (*SSD*) between the observed and expected distributions and the Harpending's raggedness index (*H*_Rag_) were mainly significant (Table 5), indicating each species is in approximate demographic equilibrium.

**Table 5:** Results of neutrality tests for regional and mismatch distribution analysis for three peony species

Species	Tajima's *D*	*p*	Fu's *Fs*	*p*	SSD	*p*	*H*_Rag_	*p*
*P. jishanensis*	1.023	0.782	8.325	1.000	0.008	0.012*	0.150	0.145
*P. qiui*	1.249	0.837	11.742	1.000	0.205	0.000**	0.383	0.000**
*P. rockii*	0.991	0.777	0.694	0.983	0.023	0.100	0.078	0.000**

Note

SSD, sum of squared deviations between observed and expected; *H*_Rag_, raggedness index.

*0.01 < *p*< 0.05; **0.001 < *p*< 0.01.

### 3.4 Nuclear microsatellite diversity and population structure

The frequency of null alleles at all loci was very low for *P. jishanensis* (0.051), *P. qiui* (0.087), and *P. rockii* (0.089), respectively. Furthermore, no evidence of linkage disequilibrium was found for pairs of nSSR loci in each species and the *F*_ST_‐outlier tests implemented in LOSITAN indicated that no locus significantly deviated from neutral expectations (Figure S3). Therefore, 22 markers were suitable for further analyses. Genotyping of 587 individuals at 22 nSSR loci resolved 182 alleles (8.273 alleles per locus on average) with allelic richness (*A*_R_) in populations ranging from 1.203 (LCZ) to 1.609 (KZL). Genetic diversity was higher in *P. qiui* (*H*_O_ = 0.444, *H*_E_ = 0.443) than in *P. jishanensis* (*H*_O_ = 0.406, *H*_E_ = 0.371) and *P. rockii* (*H*_O_ = 0.300, *H*_E_ = 0.334). *F*_IS _values were negative for *P. jishanensis* (−0.312) and *P. qiui* (−0.015), while the *F*_IS_ value was positive but low (0.098) for *P. rockii*. The number of private alleles was higher for *P. qiui* (with a mean of 1.7) than for *P. jishanensis* (with a mean of 0.2) and *P. rockii* (with a mean of 0.7; Table 3).

**Figure 5:**
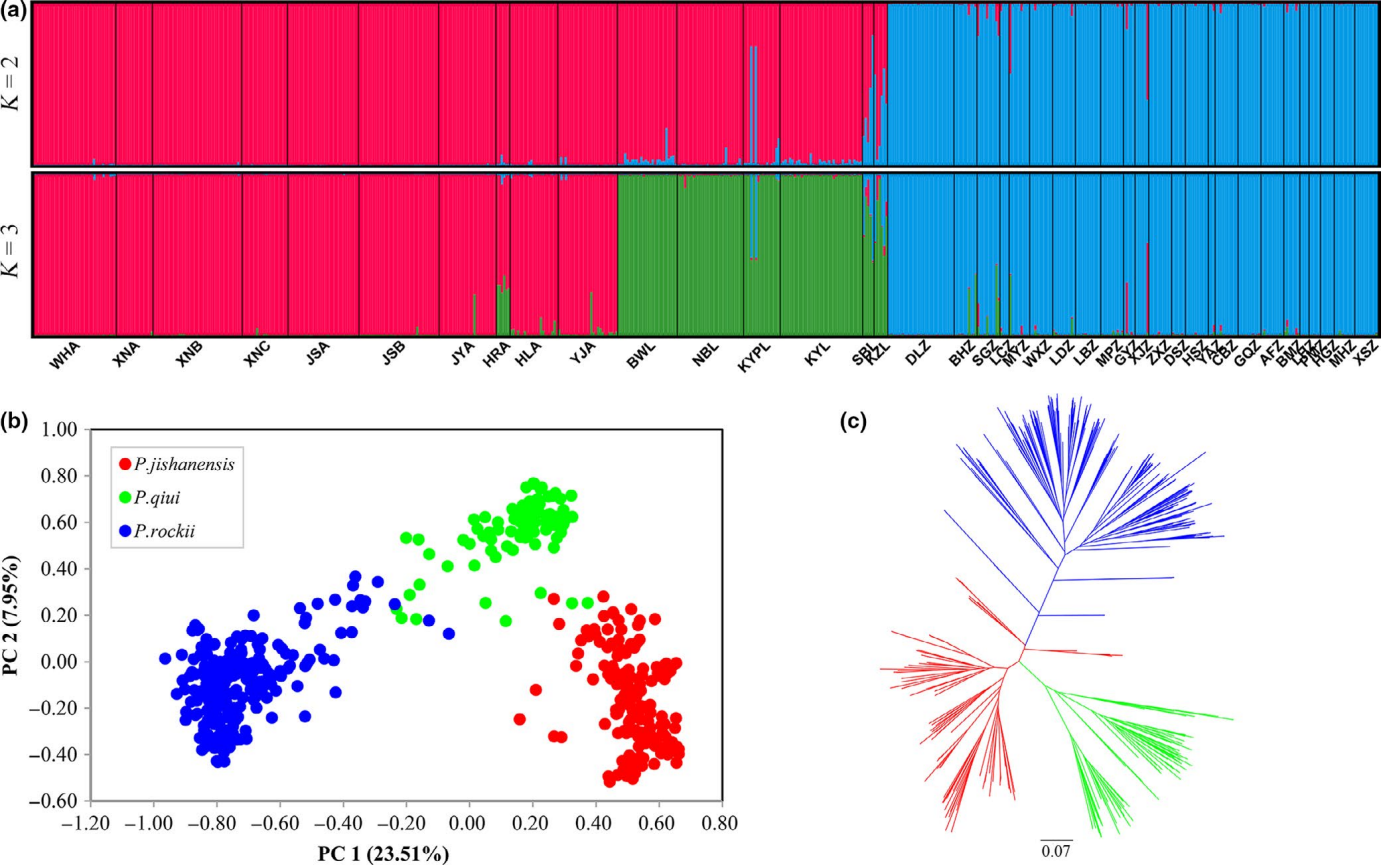
Genetic clustering of individuals of *P. jishanensis*, *P. qiui,* and *P. rockii* based on variation at 22 nSSR loci: (a) population cluster analysis using Structure (*K* = 2–3); (b) principal component analysis (PCA), and (c) unrooted neighbor‐joining tree

STRUCTURE analysis of the complete dataset indicated that the highest peak for Δ*K* was at *K* = 2, followed by *K* = 3 (Figure S4). When *K* = 2, *P. jishanensis* and *P. rockii* individuals clustered into two separate group, while some individuals of *P. qiui* were admixed (Figure 5a). Approximately 85% of the mean genetic composition of *P. qiui* came from *P. jishanensis*, while the remaining 15% came from *P. rockii*. However, when *K* = 3, *P. jishanensis*, *P. qiui,* and *P. rockii* were clearly assigned to different genetic groups, with each group representing a different species. Furthermore, the PCA of all individuals identified three groups (Figure 5b) as did the neighbor‐joining tree constructed for all samples (Figure 5c). When a separate STRUCTURE analysis for each species was conducted, two genetic groups were identified for *P. jishanensis* and for *P. qiui*, whereas three genetic groups were found for *P. rockii* (Figure S5). A similar result to STRUCTURE analysis was also supported by the PCA for each species (Figure S5).

Hierarchical AMOVA revealed significant genetic differentiation (*F*_ST_ = 0.498, *p* < 0.0001), with 20.66% of the variation partitioned among the three species and 29.23% partitioned among populations within species. For each species, AMOVA revealed the highest genetic variation partitioned among populations in *P. rockii* (42.18%) and the lowest in *P. jishanensis* (34.04%; Table 4).

**Figure 6:**
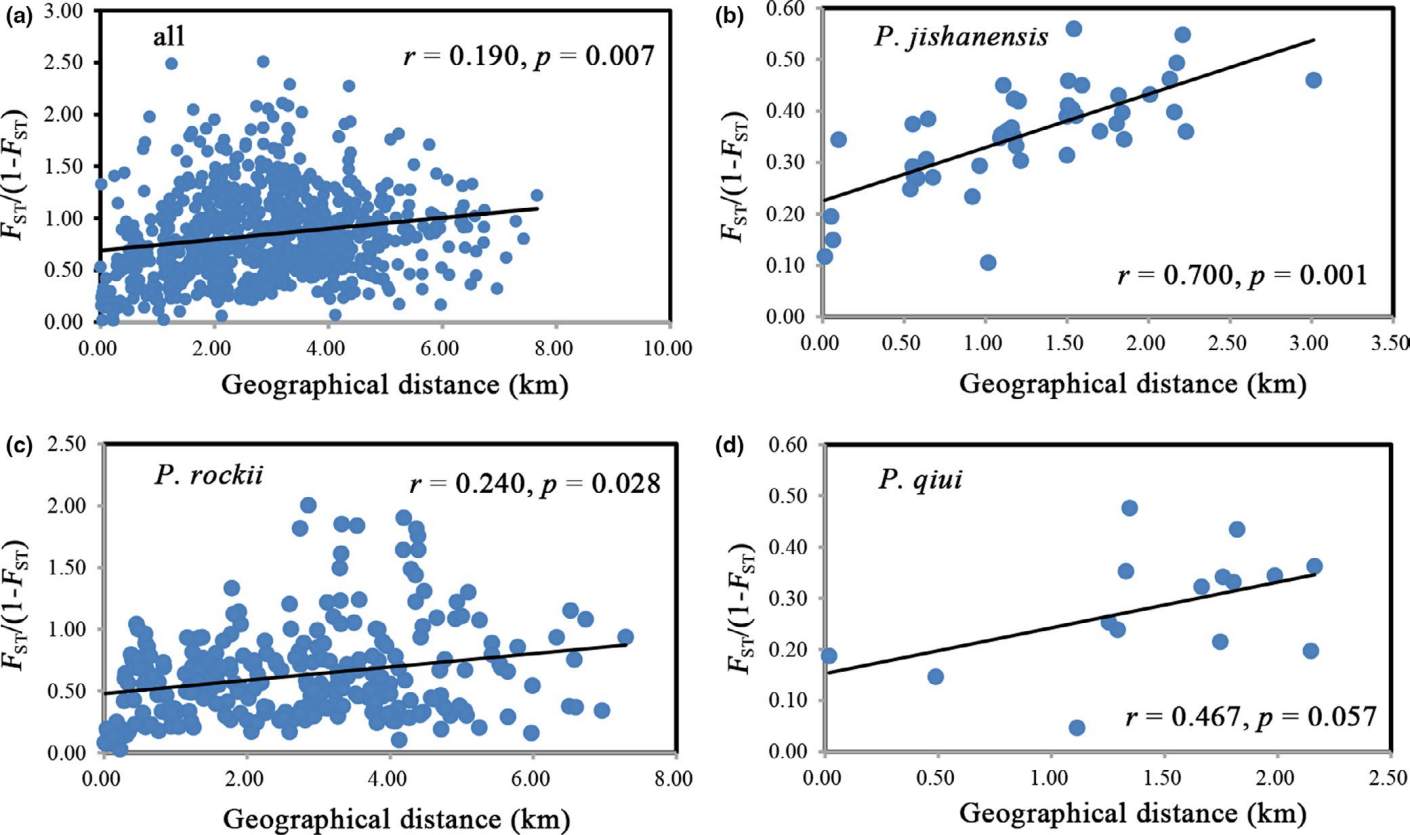
Plot of geographical distance against genetic distance for population comparisons of (a) all three species, (b) *P. jishanensis*, (c) *P. rockii,* and (d) *P. qiui*

Pairwise *F*_ST_ values for comparisons between *P. jishanensis* and *P. qiui* populations were less variable (0.301–0.569) than those between *P. jishanensis* and *P. rockii* (0.393–0.706) or *P. rockii* and *P. qiui* (0.209–0.624). For each species, pairwise *F*_ST_ values ranged from 0.105 to 0.560 in *P. jishanensis*, 0.047–0.476 in *P. qiui*, and 0.030–0.667 in *P. rockii* (Table S8). There was a significant correlation between genetic distance and geographic distance among populations of all species (*r* = 0.190, *p* = 0.007; Figure 6), *P. jishanensis* (*r* = 0.700, *p* = 0.001) and *P. rockii* (*r* = 0.240, *p* = 0.028), indicating isolation by distance. In contrast, IBD was weak for *P. qiui* (*r* = 0.467, *p* = 0.057; Figure 6).

**Figure 7:**
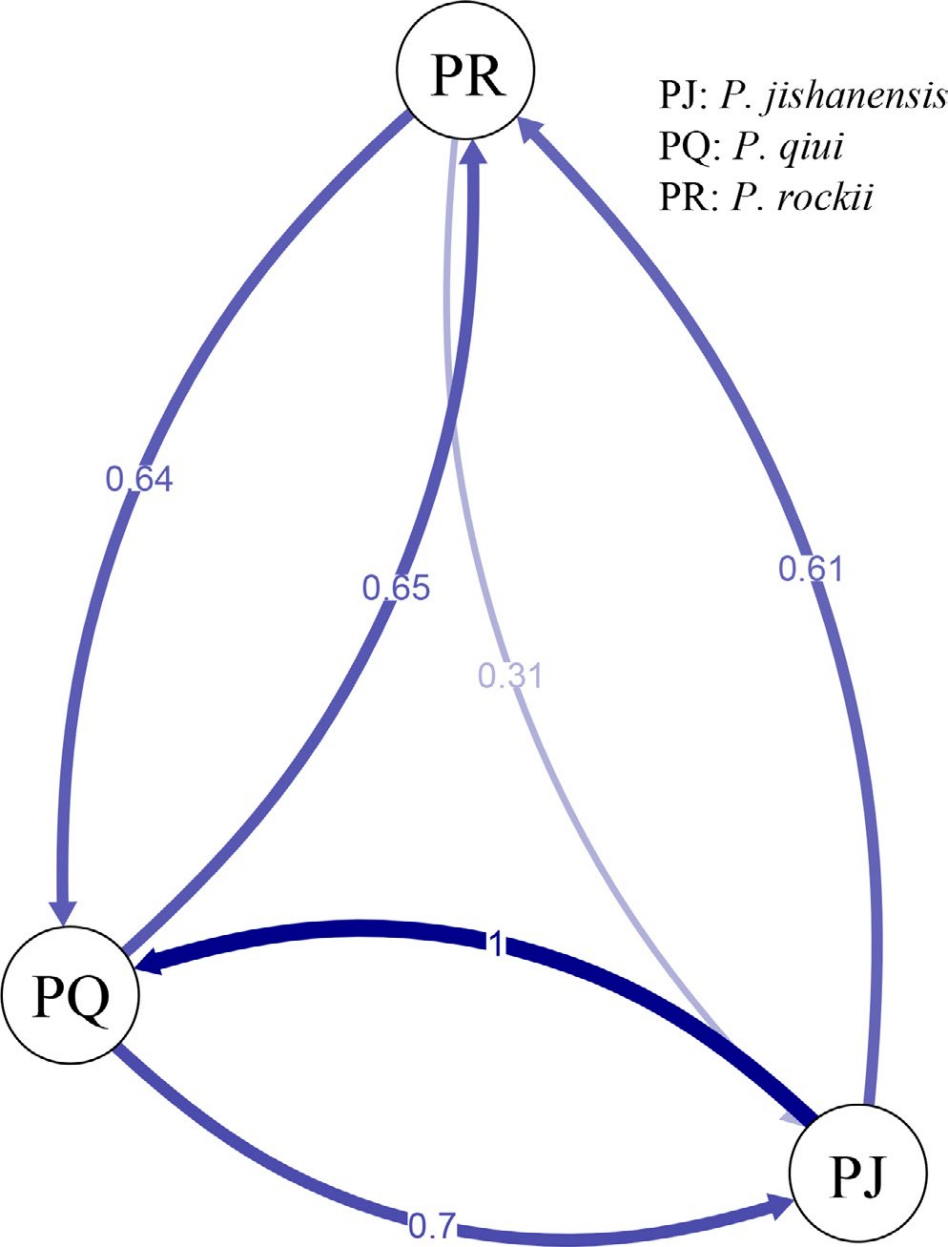
Directional relative migration networks of clustered samples from divMigrates based on *N*_m_ values

The directional relative migration networks as estimated by divMigrate (Figure 7) revealed a high degree of potential bidirectional gene flow between *P. jishanensis* and *P. qiui*, and between *P. qiui* and *P. rockii*.

### 3.5 Mode of divergence and speciation

Results of our ABC analysis of alternative model scenarios for divergence and speciation based on nSSRs data are summarized in Table 6. In step 1, scenario 1 (Figure 2a) had the highest posterior probability with a 95% confidence interval not overlapping those of the other scenarios. In step 2, the same scenario was the best supported (*PP* = 0.6104), indicating that *P. qiui* split from *P. jishanensis* at time t1, while *P. rockii* split from *P. jishanensis* at time t2 (Figure 2b). Our evaluation of confidence in scenario choice revealed low type I error and type II error, suggesting that our model choice analyses were reliable.

**Table 6:** Posterior probability of each scenario and 95% confidence intervals (CI) based on the logistic regression approach for approximate Bayesian computation analyses considering all species

Scenario	Posterior probability	95% CI	Type Ⅰ error	Type Ⅱ error
(a)				
1	0.5607	0.5555–0.5660	0.202	0.198
2	0.0062	0.0034–0.0091		
3	0.0018	0.0000– 0.0047		
4	0.0004	0.0000–0.0033		
5	0.0851	0.0817–0.0885		
6	0.3458	0.3404–0.3511		
(b)				
1	0.6104	0.6070–0.6138	0.298	0.331
2	0.3896	0.3862‐0.3930		

Results from the ABC analysis also indicated that the current population sizes of *P. jishanensis* (NJ) and *P. qiui* (NQ) decreased to 9.21 × 10^2 ^and 3.29 × 10^3^ from a larger ancestral population size of 9.52 × 10^3 ^(A2), respectively (Figure S6). In contrast, the current population size of *P. rockii* (NR) is 5.42 × 10^3^, which is slightly larger than the ancestral population size of A1 (Figure S6). Importantly, the median divergence time between *P. jishanensis* and *P. rockii* was estimated to be 50.3 Ka (95% HPD: 15.2–109 Ka), while the origin of *P. qiui* was dated to 24.3 Ka (95% HPD: 6.78.3–94.5 Ka), assuming an average generation time of 10 years. The estimated median mutation rate was 1.74 × 10^‐4^ (Table 7, Figure S6).

**Table 7:** Posterior median estimate and 95% highest posterior density interval (HPDI) for species effective population sizes, *t*1, *t*2, and μ in the best supported scenario 2 in step 2 of the ABC analysis, based on nSSRs data for three tree peony species

Parameter	NJ	NQ	NR	*t*1	*t*2	μ
Median	1,690	4,910	5,090	2,430	5,030	1.74 × 10^−4^
*q* (2.5)	558	1,680	1,920	678	1,520	1.14 × 10^−4^
*q* (97.5)	3,480	9,230	9,140	4,200	10,900	4.13 × 10^−4^

Note

*N*, effective population sizes for each species (J = *P. jishanensis*, Q = *P. qiui,* R* = P. rockii*); *t* = time in generations; μ = mutation rate.

### 3.6 Ecological niche modeling

**Figure 8:**
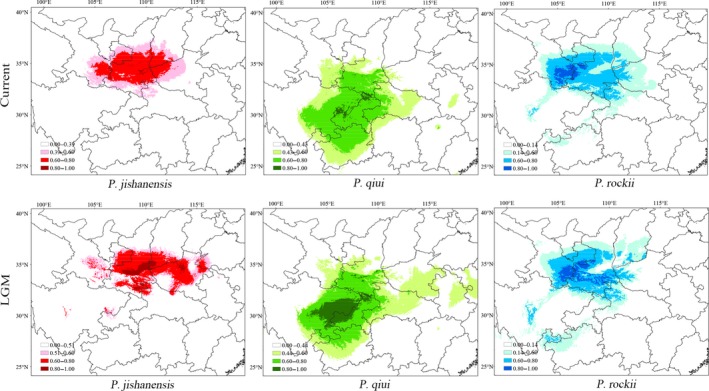
ENM predicted distributions of the three peony species at (a) currently (0 years BP) and (b) at the Last Glacial Maximum (LGM, 21,000 years BP)

Ecological niche modeling revealed that predictions for the present distributions of *P. jishanensis* and *P. rockii* were mostly congruent with observed distributions (Figure 8). However, for *P. qiui* it was evident that in some predicted areas the species is not currently present *(*e.g., northeast of Sichuan). The areas under curve values (AUCs) for the current distributions of *P. jishanensis* (0.986), *P. qiui* (0.983), and *P. rockii* (0.936) indicated good predictive model performance. Comparisons with predicted LGM distributions suggest that current distributions in areas of suitable habitat (>0.80) are much smaller for each species with loss of suitable habitat in the Qinling Mountains for *P. jishanensis* and *P. rockii,* and in the northeast of Sichuan for *P. qiui*. The climate variables that most contributed to model predictions of distributions were mean temperature of the driest quarter (Bio9, 62.2%) for *P. jishanensis,* mean temperature of the driest quarter (Bio9, 30.2%), mean diurnal range (Bio2, 18.0%) and precipitation seasonality (Bio15, 15.4%) for *P. rockii*, and mean diurnal range (Bio2, 32.1%), precipitation seasonality (Bio15, 29.1%), and precipitation of the coldest quarter (Bio19, 23.2%) for *P. qiui* (Table S3).

### 3.7 Niche divergence

Pairwise, niche space‐based multivariate tests supported significant divergence along all niche axes (*d*_b_ < *d*_n_ and *d*_n_ were significant), indicating the involvement of ecological differentiation in species differences (Table 8). Between the taxon pairs *P. jishanensis* and *P. qiui*, *P. jishanensis* and *P. rockii*, and *P. rockii and P. qiui*, two axes of the PCA jointly explained 68.8%, 68.75%, and 67.95% of the total variation, respectively. In all three of these pairwise comparisons, the reduced niche axes were primarily associated with temperature. PC1 was strongly correlated with the geographical variables (latitude, longitude, and altitude) in each pairwise comparison.

**Table 8:** Divergence on independent niche axes between species pairs *P. jishanensis*, *P. qiui,* and *P. rockii*

	*P. jishanensis* versus* P. qiui*		*P. jishanensis* versus* P. rockii*		*P. rockii* versus *P. qiui*	
	PC1	PC2	PC1	PC2	PC1	PC2
*d*_n_^a^	0.39	0.63	0.33	0.34	0.39	0.48
*d*_b _(95% null distribution)	0–0.21	0–0.16	0–0.20	0–0.15	0–0.21	0–0.15
Top‐loading variable	bio3, bio4, bio9	bio2, bio15	bio3, bio4, bio9	bio2, bio15, bio9	bio3, bio4, bio9	bio2, bio15, bio9
Percentage of variance explained	43.86	24.92	43.85	24.9	43.71	24.24
Biological interpretation	Temperature	Isothermality, temperature	Temperature	Isothermality, temperature	Temperature	Isothermality, temperature
Correlation longitude	−0.24**	0.21**	−0.24**	−0.21**	−0.24**	0.23**
Correlation latitude	−0.91**	0.06**	−0.91**	−0.06**	−0.91**	0.07**
Correlation altitude	−0.22**	−0.29**	−0.22**	0.29**	−0.22**	−0.29**

Significant niche differentiation for all PC axes (*d*_b_ < *d*_n_ and *d*_n_ is significant, *p* < 0.001).

The eight bioclimatic variables (bio2, bio3, bio4, bio5, bio9, bio13, bio15, and bio19).

** *p* < 0.001 for correlations between PC axes and geographical variables.

## 4 DISCUSSION

### 4.1 Species identification and cytoplasmic‐nuclear discordance in Paeonia

Our analyses based on morphological and nSSR data support the view that *P. jishanensis*, *P. qiui,* and *P. rockii* are distinct species. In agreement with previous morphological analyses (Zhao et al., 2008; Zhou, Pan, & Hong, 2003), our morphological analysis showed that the three peony species can be distinguished according to floral and leaf characters. In addition, they are highly divergent based on an analysis of nSSR variation. Thus, a hierarchical AMOVA of the nSSR dataset revealed significant genetic differentiation between the three species, while PCA and a neighbor‐joining tree placed *P. jishanensis*, *P. qiui,* and *P. rockii* individuals into different clusters representing the three species. Finally, ENM and multivariate niche space analysis identified significant ecological differentiation between the three species. STRUCTURE analysis of the complete dataset suggested the presence of only two genetic groups, with some *P. qiui* individuals indicated to be admixed, implying potential gene flow between *P. jishanensis* and *P. rockii*, which was also supported by the directional relative migration network as estimated by divMigrate. The latter analysis indicated high potential rates of gene flow between all three peony species. However, when K = 3, *P. qiui* individuals were assigned to a separate genetic group. A greater genetic similarity of *P. qiui* to *P. jishanensis* than to *P. rockii* was made further evident by the pairwise *F*_ST_ comparisons between species. The possibility that *P. qiui* diverged from *P. jishanensis* was supported by ABC analysis of nSSR data. Despite *P. qiui* having a more restricted distribution than *P. jishanensis* and *P. rockii*, it exhibited the highest levels of genetic diversity (at both cpDNA and nuclear microsatellite markers). Furthermore, *P. qiui* contained the highest number of private alleles in most populations based on the nSSR analysis.

In contrast to the clear separation of species according to morphological and nSSR variation, the cpDNA haplotype network showed that the 18 haplotypes resolved did not cluster by species and that *P. qiui* shared two haplotypes (H2 and H12) with *P*. *rockii*. Such cytoplasmic‐nuclear discordance in species delimitation commonly occurs in plants and has also been reported in other tree peonies of the group *P. delavayi*/*ludlowii* (Zhang et al., 2018) and may reflect the effects of incomplete lineage sorting or introgressive hybridization (Comes & Abbott, 2001; Wu & Campbell, 2005). Because the effective population size of nuclear DNA is four times that of cpDNA (Palumbi, Frank, & Hare, 2001; Schaal, Hayworth, Olsen, Rauscher, & Smith, 2010), lineage sorting should be faster for cpDNA (Guillemin et al., 2016; Qu et al., 2012). Thus, the most plausible explanation for the discordance in the present study is that hybridization and introgression occurred between the species, particularly between *P. qiui* and *P. rockii,* at times of contact in the Daba and central eastern Qinling Mountains during and/or after the LGM. There is good evidence for reticulate evolution having occurred frequently in *Paeonia* (Sang, Crawford, & Stuessy, 1995; Sang & Zhang, 1999; Yuan, 2010), and our results support this for *P. qiui* and *P. rockii* and further emphasize that biparentally inherited SSR markers are more effective than maternally inherited cpDNA markers in delimiting these species.

### 4.2 Genetic differentiation among the three tree peony species

High levels of population differentiation were observed within the three species for nSSR data and especially for cpDNA (Table 4), in comparison with the average values for plant species (Nybom, 2004 and Petit et al., 2005). Similar levels of high genetic differentiation have been reported in *P. rockii* before (cpDNA: Yuan, Cheng, & Zhou, 2011; nSSRs: Yuan et al., 2012), *P. jishanensis* (nSSRs: Xu et al., 2016) and in another tree peony, *P. delavayi* (nSSRs and plastid DNA: Zhang et al., 2018). In contrast, levels of genetic differentiation recorded among the three closely related peony species for both markers (*F*_STnSSR_ = 0.498 and *F*_STcpDNA_ = 0.929) were slightly higher than those detected between two other peony species, *P. delavayi* and *P. ludlowii* (*F*_STnSSR_ = 0.403 *and F*_STplastid DNA_ = 0.420), endemic to the Himalayan–Hengduan Mountains (Zhang et al., 2018). Although explanations for high levels of genetic differentiation have been sufficiently addressed in previous studies for *P. rockii* (Yuan et al., 2011, 2012) and *P. jishanensis* (Xu et al., 2016), relatively little is known about the factors affecting genetic divergence among the three species (Sang et al., 1995; Yuan et al., 2014).

In this study, such significant genetic differentiation indicates that despite evidence of introgressive hybridization having occurred in the past, barriers to gene flow are very strong between the species. According to our field survey, *P. rockii* is widely distributed throughout the Qinling–Daba Mountains occurring at high altitudes of 1,100–2,800 m, whereas *P. jishanensis* and *P. qiui* have more restricted distributions at lower altitudes of 750–1,700 m, to the north and south of the mountain chain, respectively (Figure 1a). Thus, currently, the species are largely isolated from each other by their different geographical distributions and also ecologically as reflected in their growth at different altitudes. On occasion, neighboring populations of the different species occur, but these are usually separated by geographic barriers, for example, high mountain ridges that are common within the diversified topography of the Qinling–Daba Mountains. Also, because tree peonies are pollinated by insects such as bees, gene flow via pollen is expected to be limited by the migratory capacity of pollinators (Luo, Pei, Pan, & Hong, 1998; Yuan et al., 2011). Similarly, gene flow via seed dispersal will be limited as seeds are normally dispersed by gravity or by rats (Hong, 2010) and consequently have a restricted dispersal radius. Thus, gene flow via pollen and seed dispersal between neighboring populations of different peony species is likely to be highly restricted, particularly if geographical features, such as mountain ridges or rivers, separate these populations. A similar observation has been made in another tree peony, *Paeonia delavayi*, distributed in the Hengduan Mountains, suggesting life‐history traits and geographic isolation have played an important role in species divergence (Zhang et al., 2018).

### 4.3 Demographic history of tree peonies and multiple refugia

DIYABC analysis of nSSR variation indicated that *P. rockii* diverged from the common ancestor of *P. jishanensis* and *P. qiui* ~50.3 Ka, corresponding closely to the Marine Isotope Stage (MIS) 3 (58–32 Ka) when China experienced two abrupt climatic warming phases during the last glacial cycle (Shi, Zhao, & Wang, 2011; Zhao, Shi, & Jie, 2011). This estimate of time of divergence between these two species is more recent than that reported previously (140–220 Ka; Yuan et al., 2014). The difference in estimated age likely results from differences in mutation rates used in the ABC computation. In the present study, we set mutation rates to 10^−4^ and 10^−3^, which is consistent with rates for EST‐SSRs in woody plants (Petit & Hampe, 2006), producing an estimated median mutation rate of 1.74 × 10^−4^. In contrast, Yuan et al. (2014) set mutation rates to 10^−3^ and 10^−2^, yielding a much higher and what we regard as less accurate median mutation rate of 1 × 10^−3^.

The most likely model of divergence and speciation also suggested that *P. qiui* diverged from *P. jishanensis* ~24.3 Ka, which is at the time of the LGM (18–25 Ka), and that its origin was not a consequence of hybridization between *P. jishanensis* and *P. rockii*. Our results indicate that Quaternary climate oscillations had an important effect in driving speciation of these sister species. Although Central China has never been directly impacted by extensive and unified ice‐sheets (Qiu et al., 2011), it nonetheless experienced climatic oscillations throughout the Quaternary with dramatic effects on the evolution and distribution of species (Harrison, Yu, Takahara, & Prentice, 2001; Qiu et al., 2011). Quaternary climate oscillations and associated environmental changes promoted range fragmentation and population isolation, thus providing opportunities for allopatric speciation (Comes, Tribsch, & Bittkau, 2008; Qiu et al., 2011). According to our field observation, *P. jishanensis* and *P. qiui* have different distributions endemic to the north and south of the Qinling mountain chain, respectively. Most species in temperate areas in Central China displayed a fragmented distribution pattern during the glacial cycles of the Quaternary (Qian & Ricklefs, 2000; Hewitt, 2000; Zhou, 2010). By comparing the relative importance of climatic variables on the distributions of the two species, we found that *P. jishanensis* and *P. qiui* show preferences for different climatic conditions. As a consequence, the geographic distributions of *P. jishanensis* and *P. qiui* likely became fragmented, thus promoting conditions for allopatric divergence among isolated populations during Quaternary climate oscillations.

Our estimates of divergence times should be treated with caution due to the wide 95% confidence intervals attached to them. In addition, vegetative reproduction by rhizomes among tree peonies may prolong generation time, causing us to underestimate divergence time (Sang et al., 1995). Nonetheless, the estimated time for divergence of all three tree peony species approximates to the most recent Pleistocene glaciation, which if correct suggests that their origin was not triggered by the rapid and massive uplift of the Qinling Mountains estimated to have occurred between 1.2 and 2.4 Ma (Yuan et al., 2012), but more likely was triggered by climate change during the late Pleistocene and the effects this had on the distribution of ancestral forms at that time.

Climate change during late Pleistocene glacial‐interglacial cycles is well known to have dramatically influenced species distributions (Comes & Kadereit, 1998; Hewitt, 1996; Klicka & Zink, 1997). During glacial periods, many temperate species in the Northern Hemisphere migrated to lower latitudes and altitudes, only to return to higher latitudes and altitudes during interglacials (Davis & Shaw, 2001; Hewitt, 1996). Reductions in genetic diversity have often been detected in higher latitude populations due to loss of such diversity during the recolonization process (Hewitt, 1996; Nason, Hamrick, & Fleming, 2002). However, in the present study, for both nSSRs and cpDNA, we did not detect significant declines in genetic diversity among populations of the three peony species with increasing latitude.

Based on cpDNA variation, none of the three species exhibited a strongly unimodal mismatch distribution or significant negative values of Tajima's *D* or Fu's *F*_S_, which suggests that they maintained relatively stable population sizes throughout the last glacial period and into the current interglacial. However, a different picture with regard to the stability of population sizes of *P. jishanensis* and *P. qiui* emerged from the ABC analyses of nSSR variation. This indicated that the population sizes of these two species were much larger during the LGM than those currently, indicating that the two species experienced historical expansions of population size throughout the LGM rather than contraction. This hypothesis is further supported by the results of ecological niche modeling, which indicated larger distribution ranges for both *P. jishanensis* and *P. qiui* at the LGM compared to currently. In contrast, the population size of *P. rockii* (NR) did not greatly alter, indicating that it may have persisted in situ and maintained a stable population size throughout the last glacial period, as suggested by the results of analyses of neutral tests and mismatch distribution of its cpDNA variation. This mode of in situ persistence during the Pleistocene glacial periods is also supported by the phylogeographic studies of other peony species, *P. ludlowii* and *P. delavayi*, in the Hengduan Mountains, although *P. delavayi* is believed to have shown both in situ persistence and retreat to refugia during these periods, Zhang et al., (2018). In contrast with the general “contraction‐expansion” scenario (Hewitt, 1996), our analyses reveal population expansion or local persistence throughout the LGM, rather than contraction. Such a phylogeographic pattern of expansion or stability during the last glacial period has also been highlighted in a few recent studies (Fan et al., 2016; Liu et al., 2013; Xie et al., 2012).

The unusual demographic history of the three tree peony species might be partly explained by the climatic characteristics of the Qinling–Daba Mountains. The east–west orientation of this long mountain range in central China forms a natural division between the current northern temperate and subtropical regions (Qian & Ricklefs, 1999, 2000). Topographically diverse landscapes in the Qinling–Daba Mountains can buffer regional climate variability and therefore create stable climatic conditions (Hewitt, 2004; Tang et al., 2018; Tzedakis, Lawson, Frogley, & Hewitt, 2002) for these peony species to maintain large population sizes in this region during the last glacial period and current interglacial, respectively.

In general, refugia are characterized by climatic stability allowing species to survive through glacial and/or interglacial periods (Tang et al., 2018; Tzedakis et al., 2002). In addition, populations in refugia usually harbor not only relatively higher genetic diversity but also haplotypic uniqueness (Petit et al., 2003; Zhou et al., 2010). In our study, as revealed by our analysis of nSSR and cpDNA variation, potential refugia existed in the western Qinling Mountains for *P. rockii* (including the locations of populations MYZ, MPZ, ZXZ, MHZ, LBZ, WXZ), in the eastern part of Daba Mountains for *P. rockii* and *P. qiui* (locations of populations BHZ, DLZ, KZL, SBL), and in the northern Qinling Mountains for *P. jishanensis* (location of population JYA) during the last ice age and into the Holocene. In addition, Shennongjia probably was the main refugium, because in this region population SBL harbors the highest number of private alleles. Indeed, the stable refugia identified for *P. rockii* are consistent with previous finding that populations of *P. rockii* located in the western Qinling Mountains harbored relatively higher genetic diversity as revealed by both cpDNA and nSSR markers (Yuan et al., 2011, 2012). Our results therefore support the hypothesis that the Qinling–Daba Mountains with a variable and complex geography could provide stable conditions for species to survive in this region, therefore fostering the accumulation of genetic diversity during the LGM (Tzedakis et al., 2002). The Qinling–Daba Mountains have long been regarded as refugia for fauna and flora during the Quaternary (Dong et al., 2011; Ying, 1994), as indicated by other studies on plants *Cathaya argyrophylla* (Wang & Ge, 2006), *Saruma henryi* (Zhou et al., 2010), and members of the *Chrysanthemum indicum* complex (Li et al., 2014), and also in some animals, for example, *Alcippe morrisonia* (Song et al., 2009) and *Feirana taihangnica* (Wang, Jiang, Xie, & Li, 2013). In the case of *Paeonia jishanensis,* the possible refugial population (JYA) is located in the Taihang Mountains of the northern Qinling range, which is also believed to have served as a refugium for the plants *Opisthopappus longilobus* and *O. taihangensis* at the time of the LGM (Wang & Yan, 2014). In summary, our study suggests that multiple refugia occurred in Qinling–Daba Mountains, which allowed the three peony species to persist in situ during the Pleistocene glaciations. The current distribution pattern of refugia is similar to that of other regional endemics including *Paeonia delavayi* distributed in Hengduan Mountains (Zhang et al., 2018) and temperate plants in Central and South China (Qiu et al., 2011) such as *Castanopsis tibetana* and *Schima superba* (Fan et al., 2016), with multiple refugia sustained across their distribution ranges. Stable climate refugia located in Qinling–Daba Mountains have long been recognized as likely centers of plant endemism in Central China (López‐Pujol et al., 2011; Ying, 1994, 2001) and foci of speciation (Tang et al., 2018). Consequently, they should be protected against deforestation and habitat destruction in order to conserve diversity at the present time and into the future.

### 4.4 Conservation implications

Knowledge of the level and structure of genetic diversity in endangered species is an important element in designing conservation programs (Ouborg, 2010). In our study, the three peony species have maintained moderate levels of genetic diversity and high levels of population differentiation. To maintain the current genetic diversity, highest priority for in situ conservation should be given to the identified stable refugia among the three species located in the Western Qinling Mountains, the Daba Mountains, and the Taihang Mountains. Populations exhibiting high genetic diversity, such as JYA, KZL, and BHZ, should be prioritized for in situ conservation. Furthermore, priority should also be given to population SBL of *P. qiui*, which possesses the highest number of private alleles. Among the three species we have studied, *P. jishanensis* and *P. qiui* have more restricted distributions, being confined to the northern and southern Qinling–Daba Mountains than *P. rockii*. However, the distribution of *P. rockii* has recently been decreasing due to habitat destruction and genetic fragmentation (Xu et al., 2017; Yuan et al., 2012). Therefore, exploitation for medicinal use and anthropogenic habitat destruction should be strictly prohibited to preserve the remnant populations of these species.

In addition, because ex situ conservation can be critical to protecting endangered species (Heywood & Iriondo, 2003), we advise the establishment of a germplasm resource nursery from seeds and rhizomes collected from populations of *P. jishanensis* and *P. qiui* that harbor high levels of genetic diversity.

## CONFLICT OF INTEREST

None declared.

## AUTHOR CONTRIBUTIONS

X‐XXu, J‐FM, and F‐YC conceived the overall study. X‐XXu performed the experiments. X‐XXu, Y‐QS, X‐GH, K‐HJ, and L‐PP analyzed the data. X‐XXu, J‐FM, F‐YC, and RA wrote the manuscript. All authors read and approved the final manuscript.

## Data Availability

The three cpDNA sequences have been deposited in GenBank (accessions numbers KY200296 and KY200338).
