# Supplementary material for: Late Pleistocene speciation of three closely related tree peonies endemic to the Qinling–Daba Mountains, a major glacial refugium in Central China
Source: Ecol Evol. 2019 Jun 17;9(13):7528–48. doi: 10.1002/ece3.5284 (PMC6635923; doi:10.1002/ece3.5284)
Supplement: Supplementary file 1 [file ECE3-9-7528-s001.docx]

**1 SUPPLEMENTARY DATA**

**Figure S1** Distributions of chloroplast DNA haplotypes and haplotype network: (A) the distributions of the 18 haplotypes across all sampled populations and (B) the network of relationships between the 18 haplotypes. Circle sizes are proportional to the number of samples each haplotype.

**Figure S2** Mismatch distributions of cpDNA established for the three peony species. (A) *P. jishanensis*; (B) *P. qiui*; (C) *P. rockii*. The blue and red lines represent the observed and expected mismatch distributions, respectively.

**Figure S3** Test for selection on SSR loci. Red, gray and yellow areas represent positive selection, neutral selection and balancing selection, respectively.

**Figure S4** Bayesian inference of the optimal number of groups (*K*) obtained by STRUCTURE relative to *P. jishanensis*, *P. rockii* and *P. qiui*: (A) The likelihood for *K* from 1 to 20 and (B) the values of Δ*K*.

**Figure S5** Genetic clustering of individuals of (A) *P. jishanensis*, (B) *P. qiui* and (C) *P. rockii* based on variation at 22 nSSR loci defined by Structure and Principal component analysis (PCA).

**Figure S6** Results of DIYABC analyses based on nSSR markers. (A) six scenarios for relationships of three peony species, regarding the variation in population size and the split and admixture events. A-H: the Posterior Probability of estimates of the parameters; I: model checking for the selected scenario 1. (B) two competing scenarios based on the scenario that NQ is split from PJ. A-F: the Posterior Probability of estimates of the parameters for the best-supported scenario 1; G: model checking for the selected scenario 1.

**Table S1** Information of 22 polymorphic EST-SSR markers used in this study

**Table S2A** The morphological characters, types and their codes

**Table S2B** The data matrix of 39 individuals and 13 morphological characters used in PCA

**Table S3** The percent contribution of 8 bioclimatic variables and the area under the receiver operating characteristic curve (AUC) in species distribution modeling (ENM).

**Table S4** Eight variables below were used in ENM test

**Table S5** The multiple-range test for 13 morphological characters among *P. jishanensis*, *P. qiui* and *P. rockii*

**Table S6** Eigenvector contributions (PC) of each character from each niche axe of component analyses based on all morphological characters among *P. jishanensis*, *P. qiui* and *P. rockii*

**Table S7** Chloroplast DNA sequence polymorphisms detected in three fragments, identifying 18 haplotypes

**Table S8** Pairwise *F*_ST_ values of all *P. jishanensis*, *P. qiui* and *P. rockii* populations based on nSSR variation.

**2 SUPPLEMENTARY FIGURES AND TABLES**


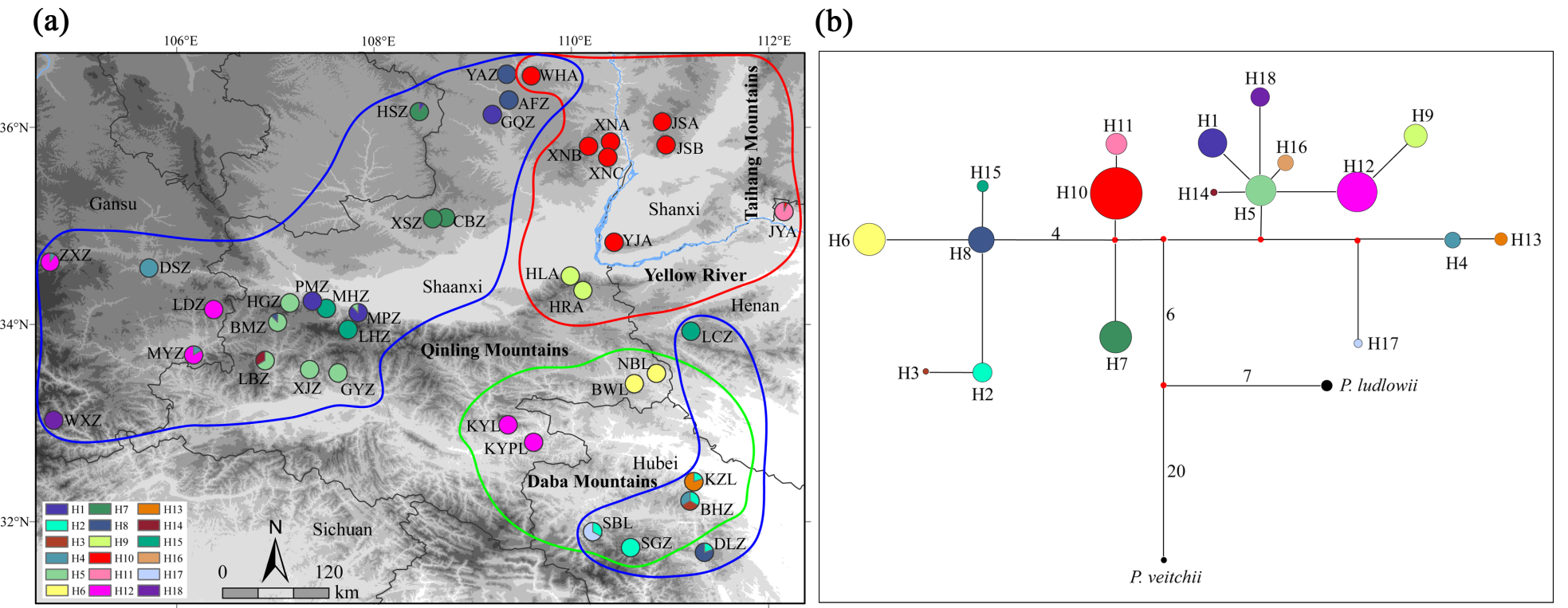


**A**

**B**

**Figure S1** Distributions of chloroplast DNA haplotypes and haplotype network: (A) the distributions of the 18 haplotypes across all sampled populations and (B) the network of relationships between the 18 haplotypes. Circle sizes are proportional to the number of samples each haplotype.

**
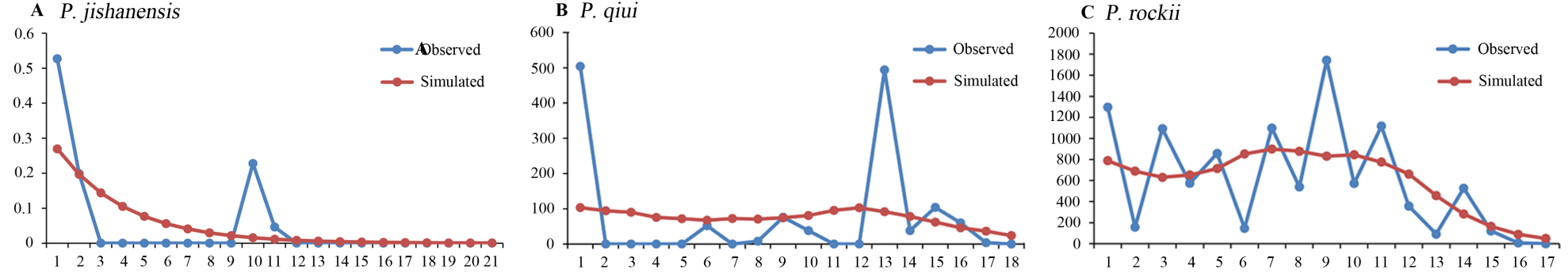
**

**Figure S2** Mismatch distributions of cpDNA established for the three peony species. (A) *P. jishanensis*; (B) *P. qiui*; (C) *P. rockii*. The blue and red lines represent the observed and expected mismatch distributions, respectively.

**
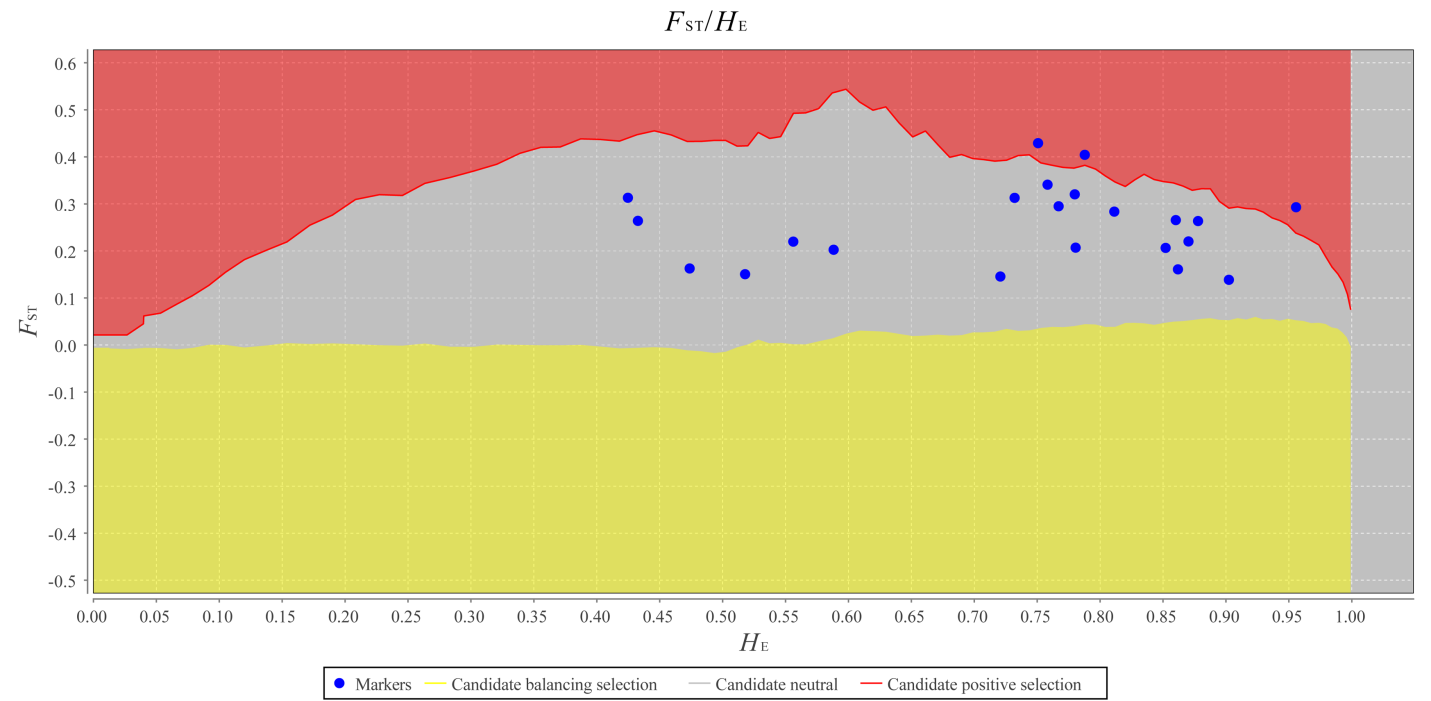
**

**Figure S3** Test for selection on SSR loci. Red, gray and yellow areas represent positive selection, neutral selection and balancing selection, respectively.


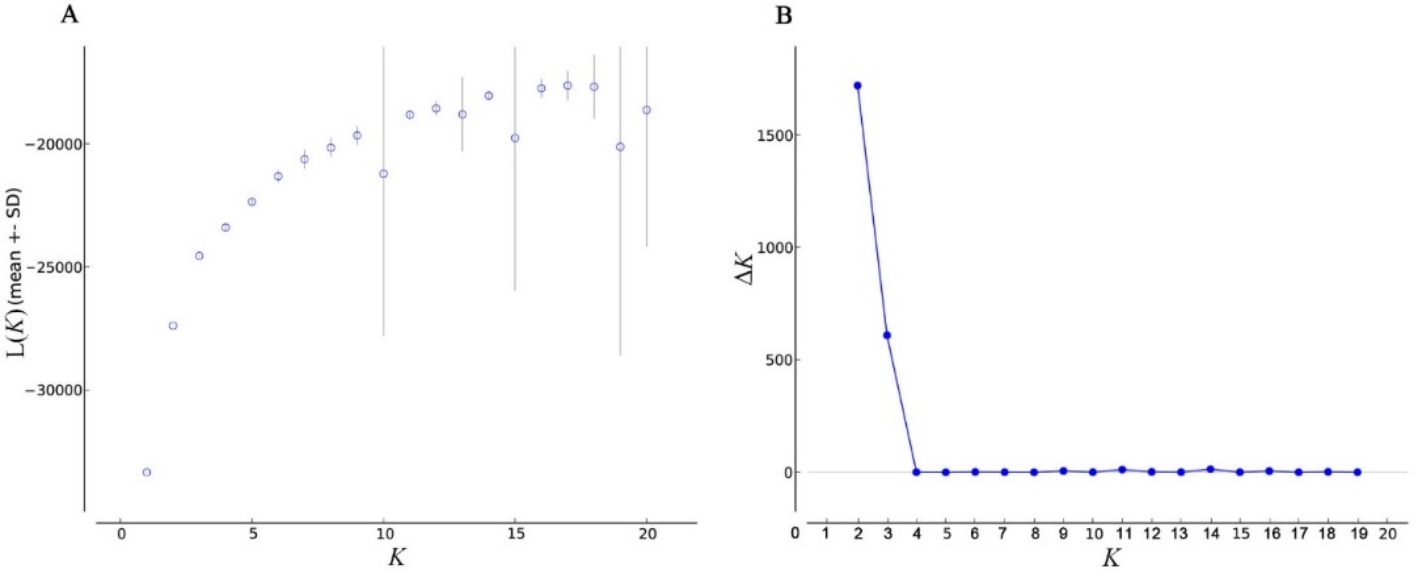


**Figure S4** Bayesian inference of the optimal number of groups (*K*) obtained by STRUCTURE relative to *P. jishanensis*, *P. rockii* and *P. qiui*: (A) The likelihood for *K* from 1 to 20 and (B) the values of Δ*K*.


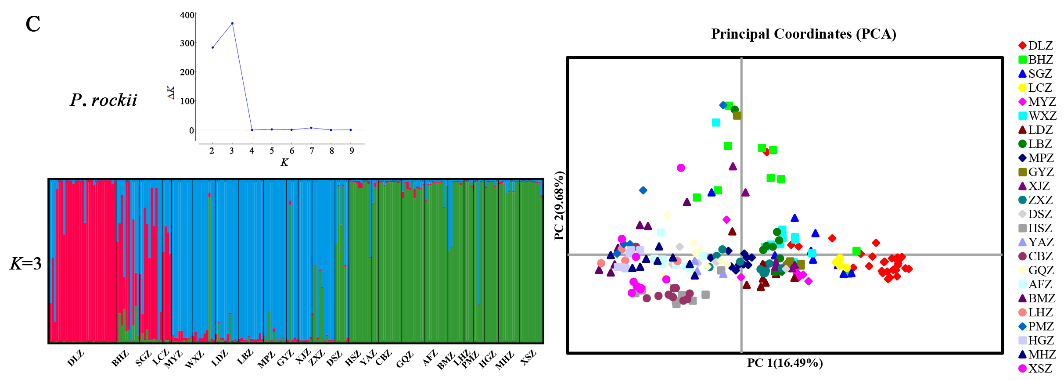

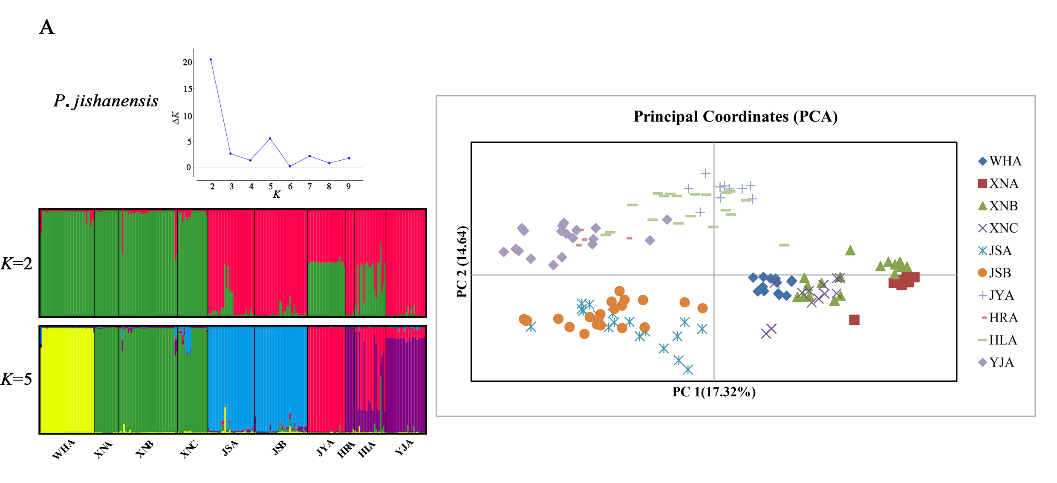

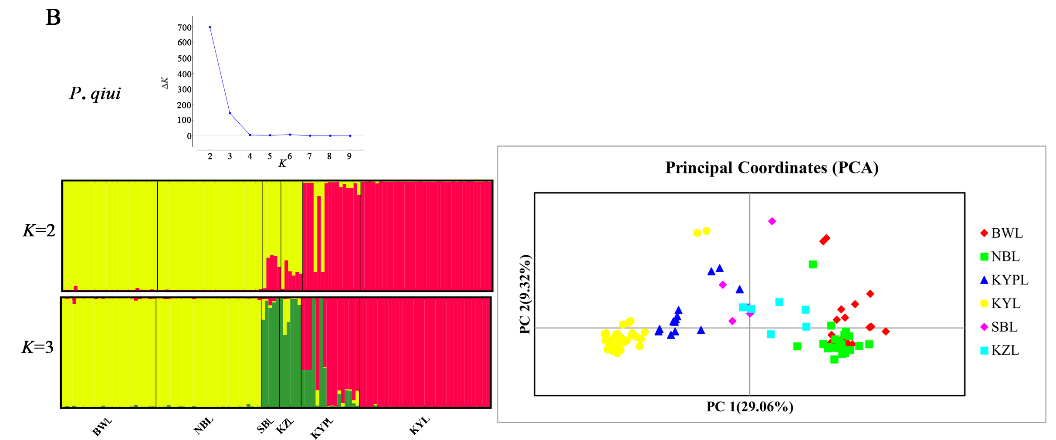
**F****igure S5** Genetic clustering of individuals of (A) *P. jishanensis*, (B) *P. qiui* and (C) *P. rockii* based on variation at 22 nSSR loci defined by Structure and Principal component analysis (PCA).


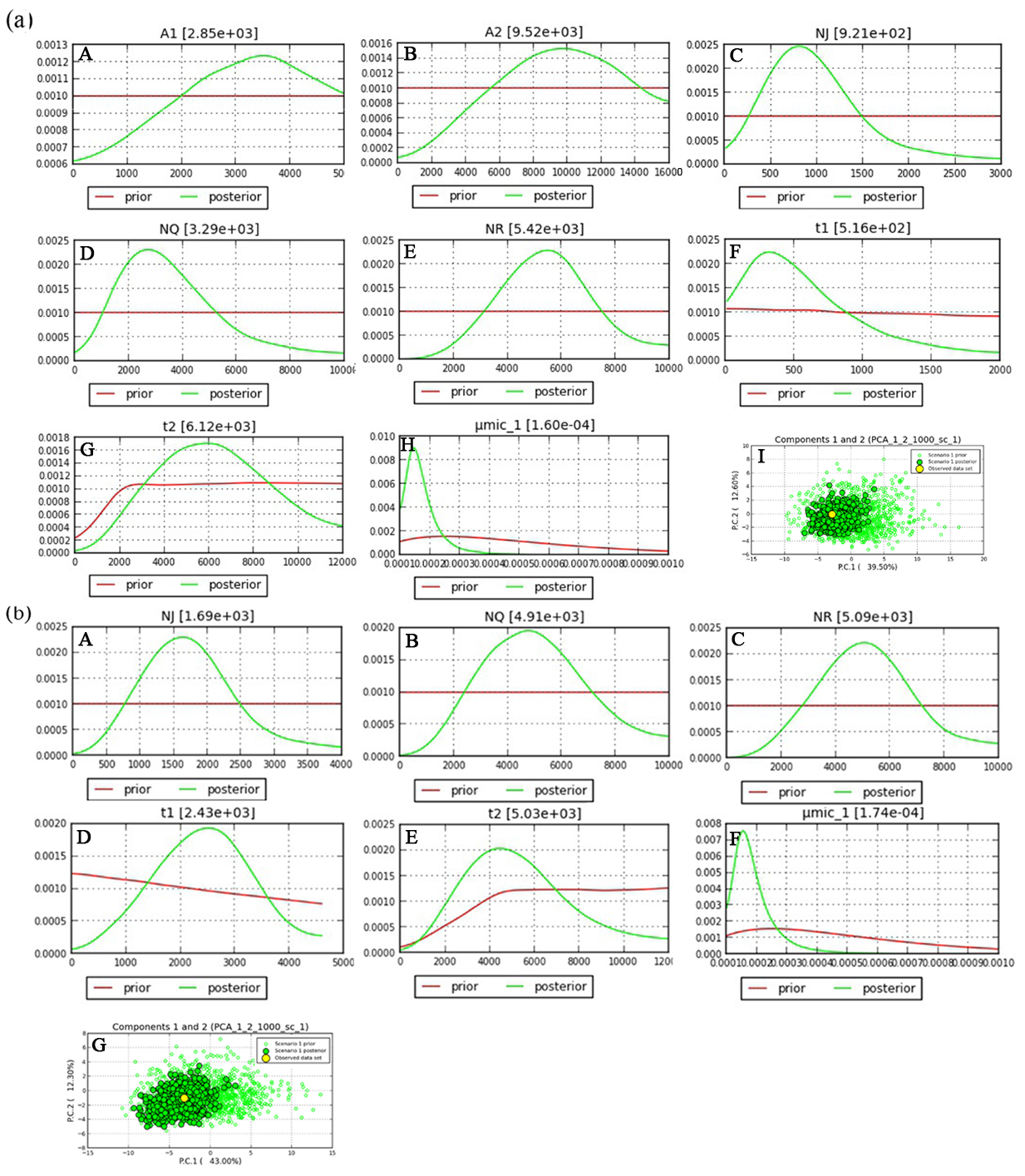


**(A)**

**(B)**

**Figure S6** Results of DIYABC analyses based on nSSR markers. (A) six scenarios for relationships of three peony species, regarding the variation in population size and the split and admixture events. A-H: the Posterior Probability of estimates of the parameters; I: model checking for the selected scenario 1. (B) two competing scenarios based on the scenario that NQ is split from PJ. A-F: the Posterior Probability of estimates of the parameters for the best-supported scenario 1; G: model checking for the selected scenario 1.

**Table S1** Information of 22 polymorphic EST-SSR markers used in this study

| Locus | Primer sequence | Ta (℃) | Expected size (bp) | Repeat Motif | GenBank Accession Number |
| --- | --- | --- | --- | --- | --- |
| PS004 | F: GTGCTTAGCCTCTAATCTG | 50.5 | 274 | (GA)_8_ | GBGY01000004 |
|  | R: CTTTGCTCCAAGTCTGTC |  |  |  |  |
| PS030 | F: ACCCTCCACCACCATCTT  R: TACTCCATCTCGTGACCC | 57 | 237 | (CT)_7_ | GBGY01000030 |
| PS047 | F: AGACGACGAGCAAAGATAT | 54 | 126 | (TC)_8_ | GBGY01000047 |
|  | R: AAAGGGCAAGATTGGAAAT |  |  |  |  |
| PS061 | F: CTCCTCCAACATTGACCC | 57 | 154 | (TG)_8_ | GBGY01000061 |
|  | R: CACCCTCCCAAACATCTC |  |  |  |  |
| PS073 | F: GTCGGTGAATGAAGGGTT | 53.5 | 269 | (AG)_6_ | GBGY01000073 |
|  | R: ATTTCTGGTCAATGTGGC |  |  |  |  |
| PS074 | F: TGCCTTGCTCCTCCTTGT | 57 | 236 | (CT)_7_ | GBGY01000074 |
|  | R: CGGTTAGCCATGAATCCC |  |  |  |  |
| PS119 | F: GCAAAGACAACAGCCTCG | 57 | 289 | (CAG)_6_ | GBGY01000120 |
|  | R: CTCACCATCCAATCCCAC |  |  |  |  |
| PS149 | F: AGTCGCCTCCTACACCTC | 55.5 | 173 | (AGG)_5_ | GBGY01000152 |
|  | R: TCCGTAAAGCCCACAATAC |  |  |  |  |
| PS157 | F: CTCCCTGAACTCCCTACC  R: CTTTCTAAACAGCCAACG | 56 | 322 | (AG)_6_ | GBGY01000160 |
| PS166 | F: TTCAGTGGGCAAGACCTAC | 55 | 337 | (AT)_7_ | GBGY01000168 |
|  | R:TAGCCAATACAGAACAAACC |  |  |  |  |
| PS180 | F:CCCCGAAATGGAGGAGTC | 60 | 188 | (CT)_6_ | GBGY01000182 |
|  | R:AGGGCAGTAGCAGAAGAAAGTC |  |  |  |  |
| PS221 | F:GATACAAGGCGGAAAGTG | 56 | 301 | (AAT)_5_ | GBGY01000223 |
|  | R:AGAGTTGGGAACCAGACC |  |  |  |  |
| PS260 | F: ATTCACGCCAGTATCAAAG  R: TGTAAATGCCCATGTCTAG | 53 | 349 | (CCTGGA)_3_ | GBGY01000261 |
| PS265 | F: TTTTATGGGTCCTGTTGC  R: GAAGAGTAAGCCTTTGTCG | 54 | 290 | (ACAGCC)_4_ | GBGY01000266 |
| PS271 | F:AGAATCCACCTCCTGTCAC | 56.5 | 406 | (GGAGAA)_3_ | GBGY01000272 |
|  | R:AACCCTGCCCTAAACTAAAC |  |  |  |  |
| PS276 | F:CTGTATCCTATCGGTTCTT | 52.5 | 447 | (CCGGTG)_4_ | GBGY01000277 |
|  | R:CCTCATCTGCCTTTATCT |  |  |  |  |
| PS296 | F: CTCTTTCGCTGCCACAAC  R: CTCTGCTCTTCCCGTCTT | 57.5 | 419 | (GAAGCA)_4_ | GBGY01000296 |
| PS311 | F: AACGCCACCATCACCTTT  R: CACCTGAACTCACCCTCC | 60 | 277 | (TTC)_6_ | GBGY01000311 |
| PS335 | F:TAATCACCCAATGAGCCA | 50 | 395 | (TTCATT)_3_ | GBGY01000334 |
|  | R:CGTCGTCGCCGAATACTT |  |  |  |  |
| PS339 | F: TGAGGCAGCCAAAGAATT  R: GGCAGGTGTAGGGTATGTT | 50 | 175 | (ACAGCA)_3_ | GBGY01000338 |
| PS356 | F:TCAAGCCCAAGGTCATTC | 53 | 354 | (GTA)_7_ | GBGY01000354 |
|  | R:ACTTGCTCACCTCGCTCT |  |  |  |  |
| PS367 | F: AGACGGACGGAAATAGGG  R: ACGAGCGATCTCAACCAT | 53.5 | 265 | (TCC)_7_ | GBGY01000365 |

**Table S2A** The morphological characters, types and their codes

| Number | Character | Types | Character states and their code numbers |
| --- | --- | --- | --- |
| C01 | flower colour of petals | D | white(0);pink(1);purple red(2) |
| C02 | colour of leafs | B | green(0); flush at the base of the leafs(1) |
| C03 | colour of carpels | B | yellow or white(0);purple red(1) |
| C04 | colour of filaments | B | yellow or white(0);purple red(1) |
| C05 | colour of stigma | B | yellow or white(0);purple red(1) |
| C06 | type of compound leaves | B | bi-compound(0);tri-compound(1) |
| C07 | flare at the base of petal | B | white(0);pink(1);purple red(2) |
| C08 | stolon | B | no(0);yes(1) |
| C09 | lobed or not in tip leaflet | B | no(0);yes(1) |
| C10 | shape of tip leaflet | N | ovate to elongated-ovate(0);ovate-lanceolate(1) |
| C11 | numbers of leaflets | N |  |
| C12 | numbers of carpels | N |  |
| C13 | height of plant | N |  |

N=Numerical character; B=Binary character; D= Disordered multistate character.

**Table S2B** The data matrix of 39 individuals and 13 morphological characters used in PCA

| Individuals | Species | C01 | C02 | C03 | C04 | C05 | C06 | C07 | C08 | C09 | C10 | C11 | C12 | C13 |
| --- | --- | --- | --- | --- | --- | --- | --- | --- | --- | --- | --- | --- | --- | --- |
| HRA01 | *P. jishanensis* | 0 | 0 | 1 | 1 | 1 | 0 | 0 | 0 | 1 | 0 | 9 | 5 | 50 |
| JYA01 | *P. jishanensis* | 0 | 0 | 1 | 1 | 1 | 0 | 0 | 1 | 1 | 0 | 9 | 5 | 135 |
| JYA02 | *P. jishanensis* | 0 | 0 | 1 | 1 | 1 | 0 | 0 | 1 | 1 | 0 | 15 | 5 | 120 |
| JYA03 | *P. jishanensis* | 0 | 0 | 1 | 1 | 1 | 0 | 0 | 1 | 1 | 0 | 11 | 5 | 125 |
| JSA01 | *P. jishanensis* | 0 | 0 | 1 | 1 | 1 | 0 | 0 | 0 | 1 | 0 | 15 | 5 | 150 |
| JSA02 | *P. jishanensis* | 0 | 0 | 1 | 1 | 1 | 0 | 0 | 0 | 1 | 0 | 9 | 5 | 55 |
| JSB01 | *P. jishanensis* | 0 | 0 | 1 | 1 | 1 | 0 | 0 | 0 | 1 | 0 | 9 | 5 | 60 |
| JSB02 | *P. jishanensis* | 0 | 0 | 1 | 1 | 1 | 0 | 0 | 0 | 1 | 0 | 9 | 5 | 45 |
| WHA01 | *P. jishanensis* | 0 | 0 | 1 | 1 | 1 | 0 | 0 | 0 | 1 | 0 | 15 | 5 | 150 |
| WHA02 | *P. jishanensis* | 1 | 0 | 1 | 1 | 1 | 0 | 1 | 0 | 1 | 0 | 9 | 5 | 40 |
| WHA03 | *P. jishanensis* | 0 | 0 | 1 | 1 | 1 | 0 | 0 | 0 | 1 | 0 | 11 | 5 | 100 |
| YJA01 | *P. jishanensis* | 0 | 0 | 1 | 1 | 1 | 0 | 0 | 0 | 1 | 0 | 15 | 5 | 55 |
| YJA02 | *P. jishanensis* | 0 | 0 | 1 | 1 | 1 | 0 | 0 | 0 | 1 | 0 | 11 | 5 | 40 |
| YJA03 | *P. jishanensis* | 0 | 0 | 1 | 1 | 1 | 0 | 0 | 0 | 1 | 0 | 15 | 5 | 50 |
| KYPL01 | *P. qiui* | 1 | 1 | 1 | 1 | 1 | 0 | 0 | 0 | 1 | 0 | 9 | 3 | 85 |
| KYPL02 | *P. qiui* | 1 | 1 | 1 | 1 | 1 | 0 | 0 | 0 | 1 | 0 | 9 | 5 | 52 |
| KYPL03 | *P. qiui* | 1 | 1 | 1 | 1 | 1 | 0 | 1 | 0 | 1 | 0 | 9 | 5 | 49 |
| BWL01 | *P. qiui* | 1 | 1 | 1 | 1 | 1 | 0 | 0 | 1 | 1 | 0 | 9 | 4 | 52 |
| BWL02 | *P. qiui* | 1 | 1 | 1 | 1 | 1 | 0 | 0 | 1 | 1 | 0 | 9 | 5 | 54 |
| BWL03 | *P. qiui* | 1 | 0 | 1 | 1 | 1 | 0 | 0 | 1 | 1 | 0 | 9 | 5 | 70 |
| CBZ01 | *P. rockii* | 0 | 0 | 0 | 0 | 0 | 1 | 2 | 0 | 1 | 1 | 20 | 5 | 80 |
| XSZ01 | *P. rockii* | 0 | 0 | 0 | 0 | 0 | 1 | 2 | 0 | 1 | 1 | 21 | 5 | 120 |
| DLZ01 | *P. rockii* | 0 | 0 | 1 | 1 | 1 | 0 | 2 | 0 | 0 | 1 | 15 | 5 | 155 |
| DLZ02 | *P. rockii* | 0 | 0 | 0 | 0 | 0 | 1 | 2 | 0 | 0 | 1 | 20 | 5 | 138 |
| DLZ03 | *P. rockii* | 0 | 0 | 0 | 0 | 0 | 1 | 2 | 0 | 0 | 1 | 25 | 5 | 160 |
| GQZ01 | *P. rockii* | 0 | 0 | 0 | 0 | 0 | 0 | 2 | 0 | 1 | 0 | 15 | 5 | 100 |
| GQZ02 | *P. rockii* | 0 | 0 | 0 | 0 | 0 | 0 | 2 | 0 | 1 | 0 | 19 | 5 | 80 |
| GQZ03 | *P. rockii* | 2 | 0 | 0 | 0 | 0 | 0 | 2 | 0 | 1 | 0 | 20 | 5 | 65 |
| LCZ01 | *P. rockii* | 0 | 0 | 0 | 0 | 0 | 1 | 2 | 0 | 0 | 1 | 33 | 5 | 24 |
| LCZ02 | *P. rockii* | 0 | 0 | 0 | 0 | 0 | 1 | 2 | 0 | 0 | 1 | 34 | 5 | 22 |
| LCZ03 | *P. rockii* | 0 | 0 | 0 | 0 | 0 | 1 | 2 | 0 | 0 | 1 | 32 | 5 | 25 |
| HSZ01 | *P. rockii* | 0 | 0 | 0 | 0 | 0 | 1 | 2 | 0 | 1 | 0 | 20 | 5 | 38 |
| HSZ02 | *P. rockii* | 0 | 0 | 0 | 0 | 0 | 1 | 2 | 0 | 1 | 0 | 29 | 5 | 35 |
| HSZ03 | *P. rockii* | 0 | 0 | 0 | 0 | 0 | 1 | 2 | 0 | 1 | 0 | 33 | 5 | 37 |
| HSZ04 | *P. rockii* | 0 | 0 | 0 | 0 | 0 | 1 | 2 | 0 | 1 | 0 | 32 | 5 | 39 |
| AFZ01 | *P. rockii* | 0 | 0 | 0 | 0 | 0 | 0 | 2 | 0 | 1 | 0 | 21 | 5 | 150 |
| AFZ02 | *P. rockii* | 0 | 0 | 0 | 0 | 0 | 0 | 2 | 0 | 1 | 0 | 19 | 5 | 120 |
| AFZ03 | *P. rockii* | 1 | 0 | 0 | 0 | 0 | 0 | 2 | 0 | 1 | 0 | 15 | 5 | 125 |
| AFZ04 | *P. rockii* | 2 | 0 | 0 | 0 | 0 | 0 | 2 | 0 | 1 | 0 | 15 | 5 | 145 |

**Table S3** The percent contribution of 8 bioclimatic variables and the area under the receiver operating characteristic curve (AUC) in species distribution modeling (ENM).

| Species | n | Bio2 | Bio3 | Bio4 | Bio5 | Bio9 | Bio13 | Bio15 | Bio19 | AUC (mean ± SD) |
| --- | --- | --- | --- | --- | --- | --- | --- | --- | --- | --- |
| *P. jishanensis* | 39 | 7.8 | 3.6 | 0.4 | 0.0 | 62.2 | 12.8 | 4.1 | 9.1 | 0.986 ± 0.005 |
| *P. qiui* | 30 | 32.1 | 3.5 | 6.6 | 1.2 | 0.7 | 3.6 | 29.1 | 23.2 | 0.936 ± 0.022 |
| *P. rockii* | 58 | 18 | 3.9 | 6.6 | 3.0 | 30.2 | 12.0 | 15.4 | 10.9 | 0.983 ± 0.002 |

**Table S4** Eight variables below were used in ENM test

| Correlation | Bio 2 | Bio 3 | Bio 4 | Bio 5 | Bio 9 | Bio 13 | Bio 15 | Bio 19 |
| --- | --- | --- | --- | --- | --- | --- | --- | --- |
| Bio 2 |  | 0.306602 | 0.388814 | -0.36417 | -0.61969 | -0.73474 | 0.591569 | -0.73345 |
| Bio 3 |  |  | -0.71814 | -0.52812 | 0.22337 | 0.032832 | 0.237909 | -0.12613 |
| Bio 4 |  |  |  | 0.260343 | -0.67529 | -0.4568 | 0.21654 | 0.336819 |
| Bio 5 |  |  |  |  | 0.515428 | 0.240384 | -0.42187 | 0.643946 |
| Bio 9 |  |  |  |  |  | 0.571309 | -0.52996 | 0.643946 |
| Bio 13 |  |  |  |  |  |  | -0.26991 | 0.690206 |
| Bio 15 |  |  |  |  |  |  |  | -0.62853 |
| Bio 19 |  |  |  |  |  |  |  | 0 |

**Table S5** The multiple-range test for 13 morphological characters among *P. jishanensis*, *P. qiui* and *P. rockii*

| Traits | Species | M | mean |
| --- | --- | --- | --- |
| flower colour of petals | *P. qiui* | a | 1 ± 0 |
|  | *P. rockii* | b | 0.26 ± 0.15 |
|  | *P. jishanensis* | b | 0.07 ± 0.07 |
| colour of leafs | *P. qiui* | a | 0.83 ± 0.16 |
|  | *P. jishanensis* | b | 0 ± 0 |
|  | *P. rockii* | b | 0 ± 0 |
| colour of carpels | *P. jishanensis* | a | 1 ± 0 |
|  | *P. qiui* | a | 1 ± 0 |
|  | *P. rockii* | b | 0.05 ±0.05 |
| colour of filaments | *P. jishanensis* | a | 1 ± 0 |
|  | *P. qiui* | a | 1 ± 0 |
|  | *P. rockii* | b | 0.05 ± 0.05 |
| colour of stigma | *P. jishanensis* | a | 1 ± 0 |
|  | *P. qiui* | a | 1 ± 0 |
|  | *P. rockii* | b | 0.05 ± 0.05 |
| type of compound leaves | *P. rockii* | a | 0.58 ± 0.11 |
|  | *P. jishanensis* | b | 0 ± 0 |
|  | *P. qiui* | b | 0 ± 0 |
| flare at the base of petal | *P. rockii* | a | 2 ± 0 |
|  | *P. qiui* | b | 0.17 ± 0.17 |
|  | *P. jishanensis* | b | 0.07 ± 0.07 |
| stolon | *P. qiui* | a | 0.5 ± 0.22 |
|  | *P. jishanensis* | ab | 0.2 ± 0.11 |
|  | *P. rockii* | b | 0 ± 0 |
| lobed or not in tip leaflet | *P. jishanensis* | a | 1 ± 0 |
|  | *P. qiui* | ab | 1 ± 0 |
|  | *P. rockii* | b | 0.68 ± 0.11 |
| shape of tip leaflet | *P. rockii* | a | 0.42 ± 0.11 |
|  | *P. jishanensis* | b | 0 ± 0 |
|  | *P. qiui* | b | 0 ± 0 |
| numbers of leaflets | *P. rockii* | a | 23.05 ± 1.58 |
|  | *P. jishanensis* | b | 11.57 ± 0.74 |
|  | *P. qiui* | c | 9 ± 0 |
| numbers of carpels | *P. jishanensis* | a | 5 ± 0 |
|  | *P. rockii* | a | 5 ± 0 |
|  | *P. qiui* | b | 4.5 ± 0.34 |
| height of plant | *P. jishanensis* | a | 87.26 ± 11.62 |
|  | *P. rockii* | a | 83.93 ± 11.60 |
|  | *P. qiui* | a | 60.33 ± 5.80 |

Means with different superscript letters (a, b, c, and d) are significantly different (*P* = 0.05) in least significant difference multiple-range tests.

**Table** **S6** Eigenvector contributions (PC) of each character from each niche axe of component analyses based on all morphological characters among *P. jishanensis*, *P. qiui* and *P. rockii*

| Traits | PC1 | PC2 | PC3 | PC4 |
| --- | --- | --- | --- | --- |
| flower colour of petals | 0.11 | 0.57 | 0.07 | 0.22 |
| colour of leafs | 0.19 | 0.47 | -0.38 | 0.02 |
| colour of carpels | 0.37 | -0.21 | -0.17 | -0.05 |
| colour of filaments | 0.37 | -0.21 | -0.17 | -0.05 |
| colour of stigma | 0.37 | -0.21 | -0.17 | -0.05 |
| type of compound leaves | -0.33 | -0.02 | -0.27 | -0.21 |
| flare at the base of petal | -0.36 | 0.18 | 0.11 | 0.13 |
| stolon | 0.19 | -0.02 | -0.12 | 0.23 |
| lobed or not in tip leaflet | 0.23 | 0.21 | 0.5 | -0.23 |
| shape of tip leaflet | -0.26 | -0.19 | -0.47 | 0.24 |
| numbers of leaflets | -0.36 | 0 | -0.09 | -0.18 |
| number of carpels | -0.12 | -0.42 | 0.37 | -0.12 |
| height of plant | 0 | -0.18 | 0.22 | 0.82 |
| Proportion of Variance | 0.48 | 0.13 | 0.12 | 0.09 |
| Cumulative Proportion | 0.48 | 0.61 | 0.73 | 0.82 |

|  |
| --- |

**Table S7** Chloroplast DNA sequence polymorphisms detected in three fragments, identifying 18 haplotypes

| Haplotype | *acc*D-*psa*I | | | | | | | | | | | | |  | *pet*B-*pet*D | | | | | | | | | |  | *psb*E-*pet*L | | | | | | | | | | | | | | |
| --- | --- | --- | --- | --- | --- | --- | --- | --- | --- | --- | --- | --- | --- | --- | --- | --- | --- | --- | --- | --- | --- | --- | --- | --- | --- | --- | --- | --- | --- | --- | --- | --- | --- | --- | --- | --- | --- | --- | --- | --- |
|  | 9 | 1 | 1 | 1 | 2 | 2 | 3 | 3 | 3 | 3 | 4 | 4 | 4 |  | 6 | 6 | 6 | 8 | 1 | 1 | 1 | 1 | 1 | 1 |  | 1 | 1 | 1 | 1 | 1 | 1 | 1 | 1 | 1 | 1 | 1 | 1 | 2 | 2 | 2 |
|  | 1 | 7 | 9 | 9 | 5 | 8 | 2 | 2 | 6 | 9 | 0 | 2 | 4 |  | 2 | 4 | 5 | 3 | 0 | 0 | 0 | 1 | 1 | 3 |  | 3 | 4 | 4 | 4 | 5 | 6 | 6 | 6 | 6 | 7 | 7 | 9 | 0 | 0 | 0 |
|  |  | 9 | 3 | 5 | 4 | 0 | 6 | 7 | 4 | 6 | 1 | 4 | 9 |  | 2 | 0 | 5 | 2 | 0 | 4 | 6 | 6 | 8 | 3 |  | 5 | 1 | 2 | 6 | 4 | 0 | 3 | 5 | 5 | 0 | 6 | 2 | 1 | 1 | 6 |
|  |  |  |  |  |  |  |  |  |  |  |  |  |  |  |  |  |  |  | 3 | 9 | 3 | 5 | 2 | 6 |  | 6 | 7 | 0 | 7 | 2 | 2 | 2 | 4 | 5 | 1 | 9 | 4 | 4 | 5 | 5 |
| H 1 | C | C | G | A | C | T | C | G | G | A | C | T | 1 |  | A | G | C | C | G | C | G | A | G | C |  | T | C | C | G | A | C | C | C | A | G | G | A | T | A | G |
| H 2 | T | T | G | A | A | C | C | G | G | A | T | C | 1 |  | C | A | T | C | T | C | G | A | G | T |  | T | C | C | G | A | C | C | C | A | A | G | C | T | A | G |
| H 3 | T | T | G | A | A | C | C | G | T | A | T | C | 1 |  | C | A | T | C | T | C | G | A | G | T |  | T | C | C | G | A | C | C | C | A | A | G | C | T | A | G |
| H 4 | C | C | G | A | A | C | C | G | G | G | C | T | 1 |  | A | G | C | T | T | C | G | A | G | C |  | T | C | C | G | A | C | C | A | A | G | G | A | T | A | T |
| H 5 | C | C | G | A | C | C | C | G | G | A | C | T | 1 |  | A | G | C | C | T | C | G | A | G | C |  | T | C | C | G | A | C | C | C | A | G | G | A | T | A | G |
| H 6 | T | T | G | A | A | C | C | G | G | A | T | C | 1 |  | A | G | C | C | T | C | G | A | G | T |  | T | T | C | G | A | C | C | C | A | A | G | C | G | A | G |
| H 7 | T | C | G | A | A | C | C | G | G | A | C | C | 1 |  | A | G | C | C | T | C | A | A | G | C |  | T | C | C | A | A | C | C | C | A | G | G | C | T | A | G |
| H 8 | T | T | G | A | A | C | C | G | G | A | T | C | 1 |  | A | G | C | C | T | C | G | A | G | T |  | T | C | C | G | A | C | C | C | A | A | G | C | T | A | G |
| H 9 | C | C | G | A | C | C | C | G | G | A | C | T | 1 |  | A | G | C | C | T | T | G | A | A | C |  | T | C | C | G | A | T | T | C | A | G | G | A | T | A | G |
| H 10 | T | C | G | A | A | C | C | G | G | A | C | C | 1 |  | A | G | C | C | T | C | G | A | G | C |  | T | C | C | G | A | C | C | C | C | G | G | C | T | A | G |
| H 11 | T | C | G | A | A | C | C | G | G | A | C | C | 1 |  | A | G | C | C | T | C | G | A | G | C |  | T | C | C | G | C | C | C | C | C | G | G | C | T | A | G |
| H 12 | C | C | G | A | C | C | C | G | G | A | C | T | 1 |  | A | G | C | C | T | T | G | A | G | C |  | T | C | C | G | A | T | C | C | A | G | G | A | T | A | G |
| H 13 | C | C | G | A | A | C | C | G | G | G | C | T | 1 |  | A | G | C | T | T | C | G | A | G | C |  | T | C | C | G | A | C | C | A | A | G | A | A | T | A | T |
| H 14 | C | C | G | A | C | C | C | G | G | A | C | T | 1 |  | A | G | C | C | T | C | G | A | G | C |  | T | C | T | G | A | C | C | C | A | G | G | A | T | A | G |
| H 15 | T | T | G | A | A | C | C | G | G | A | T | C | 0^a^ |  | A | G | C | C | T | C | G | A | G | T |  | T | C | C | G | A | C | C | C | A | A | G | C | T | A | G |
| H 16 | C | C | G | A | C | C | C | G | G | A | C | T | 1 |  | A | G | C | C | T | C | G | A | G | C |  | T | C | C | G | A | C | C | C | A | G | G | A | T | C | G |
| H 17 | C | C | A | G | A | C | C | A | G | G | C | T | 1 |  | A | G | C | T | T | C | G | A | G | C |  | G | C | C | G | A | C | C | C | A | G | G | A | T | A | G |
| H 18 | C | C | G | A | C | C | G | G | G | A | C | T | 1 |  | A | G | C | C | T | C | G | C | G | C |  | T | C | C | G | A | C | C | C | A | G | G | A | T | A | G |

Numbers 1/0 in sequences denote presence/absence of length polymorphism. ^a^ATGGAGTC

**Table S8** Pairwise *F*_ST_ values of all *P. jishanensis*, *P. qiui* and *P. rockii* populations based on nSSR variation.

|  | WHA | XNA | XNB | XNC | JSA | JSB | JYA | HRA | HLA | YJA | BWL | NBL | KYPL | KYL | SBL | KZL | DLZ | BHZ | SGZ | LCZ | MYZ | WXZ | LDZ | LBZ | MPZ | GYZ | XJZ | ZXZ | DSZ | HSZ | YAZ | CBZ | GQZ |  | AFZ | BMZ | LHZ | PMZ | HGZ | MHZ | XSZ |
| --- | --- | --- | --- | --- | --- | --- | --- | --- | --- | --- | --- | --- | --- | --- | --- | --- | --- | --- | --- | --- | --- | --- | --- | --- | --- | --- | --- | --- | --- | --- | --- | --- | --- | --- | --- | --- | --- | --- | --- | --- | --- |
| WHA | 0.000 |  |  |  |  |  |  |  |  |  |  |  |  |  |  |  |  |  |  |  |  |  |  |  |  |  |  |  |  |  |  |  |  |  |  |  |  |  |  |  |  |
| XNA | 0.420 | 0.000 |  |  |  |  |  |  |  |  |  |  |  |  |  |  |  |  |  |  |  |  |  |  |  |  |  |  |  |  |  |  |  |  |  |  |  |  |  |  |  |
| XNB | 0.304 | 0.118 | 0.000 |  |  |  |  |  |  |  |  |  |  |  |  |  |  |  |  |  |  |  |  |  |  |  |  |  |  |  |  |  |  |  |  |  |  |  |  |  |  |
| XNC | 0.349 | 0.195 | 0.150 | 0.000 |  |  |  |  |  |  |  |  |  |  |  |  |  |  |  |  |  |  |  |  |  |  |  |  |  |  |  |  |  |  |  |  |  |  |  |  |  |
| JSA | 0.345 | 0.384 | 0.306 | 0.271 | 0.000 |  |  |  |  |  |  |  |  |  |  |  |  |  |  |  |  |  |  |  |  |  |  |  |  |  |  |  |  |  |  |  |  |  |  |  |  |
| JSB | 0.348 | 0.375 | 0.292 | 0.269 | 0.105 | 0.000 |  |  |  |  |  |  |  |  |  |  |  |  |  |  |  |  |  |  |  |  |  |  |  |  |  |  |  |  |  |  |  |  |  |  |  |
| JYA | 0.460 | 0.430 | 0.376 | 0.397 | 0.423 | 0.398 | 0.000 |  |  |  |  |  |  |  |  |  |  |  |  |  |  |  |  |  |  |  |  |  |  |  |  |  |  |  |  |  |  |  |  |  |  |
| HRA | 0.548 | 0.560 | 0.403 | 0.450 | 0.390 | 0.332 | 0.493 | 0.000 |  |  |  |  |  |  |  |  |  |  |  |  |  |  |  |  |  |  |  |  |  |  |  |  |  |  |  |  |  |  |  |  |  |
| HLA | 0.462 | 0.459 | 0.314 | 0.391 | 0.411 | 0.360 | 0.360 | 0.344 | 0.000 |  |  |  |  |  |  |  |  |  |  |  |  |  |  |  |  |  |  |  |  |  |  |  |  |  |  |  |  |  |  |  |  |
| YJA | 0.432 | 0.450 | 0.354 | 0.368 | 0.294 | 0.233 | 0.361 | 0.248 | 0.272 | 0.000 |  |  |  |  |  |  |  |  |  |  |  |  |  |  |  |  |  |  |  |  |  |  |  |  |  |  |  |  |  |  |  |
| BWL | 0.560 | 0.552 | 0.478 | 0.490 | 0.458 | 0.428 | 0.481 | 0.434 | 0.459 | 0.401 | 0.000 |  |  |  |  |  |  |  |  |  |  |  |  |  |  |  |  |  |  |  |  |  |  |  |  |  |  |  |  |  |  |
| NBL | 0.510 | 0.510 | 0.443 | 0.470 | 0.415 | 0.365 | 0.438 | 0.352 | 0.394 | 0.302 | 0.146 | 0.000 |  |  |  |  |  |  |  |  |  |  |  |  |  |  |  |  |  |  |  |  |  |  |  |  |  |  |  |  |  |
| KYPL | 0.470 | 0.469 | 0.410 | 0.374 | 0.381 | 0.338 | 0.413 | 0.339 | 0.392 | 0.301 | 0.353 | 0.332 | 0.000 |  |  |  |  |  |  |  |  |  |  |  |  |  |  |  |  |  |  |  |  |  |  |  |  |  |  |  |  |
| KYL | 0.557 | 0.512 | 0.445 | 0.422 | 0.470 | 0.407 | 0.481 | 0.421 | 0.455 | 0.384 | 0.476 | 0.434 | 0.187 | 0.000 |  |  |  |  |  |  |  |  |  |  |  |  |  |  |  |  |  |  |  |  |  |  |  |  |  |  |  |
| SBL | 0.569 | 0.469 | 0.386 | 0.389 | 0.411 | 0.376 | 0.432 | 0.467 | 0.443 | 0.358 | 0.322 | 0.344 | 0.215 | 0.341 | 0.000 |  |  |  |  |  |  |  |  |  |  |  |  |  |  |  |  |  |  |  |  |  |  |  |  |  |  |
| KZL | 0.506 | 0.460 | 0.384 | 0.375 | 0.360 | 0.307 | 0.400 | 0.387 | 0.391 | 0.302 | 0.253 | 0.239 | 0.197 | 0.363 | 0.047 | 0.000 |  |  |  |  |  |  |  |  |  |  |  |  |  |  |  |  |  |  |  |  |  |  |  |  |  |
| DLZ | 0.651 | 0.566 | 0.510 | 0.563 | 0.577 | 0.561 | 0.596 | 0.693 | 0.620 | 0.602 | 0.597 | 0.589 | 0.573 | 0.624 | 0.604 | 0.550 | 0.000 |  |  |  |  |  |  |  |  |  |  |  |  |  |  |  |  |  |  |  |  |  |  |  |  |
| BHZ | 0.560 | 0.440 | 0.393 | 0.419 | 0.450 | 0.422 | 0.440 | 0.469 | 0.453 | 0.417 | 0.429 | 0.436 | 0.319 | 0.462 | 0.209 | 0.217 | 0.243 | 0.000 |  |  |  |  |  |  |  |  |  |  |  |  |  |  |  |  |  |  |  |  |  |  |  |
| SGZ | 0.575 | 0.479 | 0.430 | 0.461 | 0.481 | 0.457 | 0.501 | 0.521 | 0.504 | 0.490 | 0.470 | 0.467 | 0.370 | 0.479 | 0.347 | 0.308 | 0.320 | 0.152 | 0.000 |  |  |  |  |  |  |  |  |  |  |  |  |  |  |  |  |  |  |  |  |  |  |
| LCZ | 0.683 | 0.601 | 0.520 | 0.563 | 0.566 | 0.531 | 0.599 | 0.706 | 0.609 | 0.555 | 0.556 | 0.541 | 0.456 | 0.544 | 0.486 | 0.423 | 0.475 | 0.292 | 0.269 | 0.000 |  |  |  |  |  |  |  |  |  |  |  |  |  |  |  |  |  |  |  |  |  |
| MYZ | 0.597 | 0.495 | 0.439 | 0.467 | 0.493 | 0.467 | 0.492 | 0.546 | 0.510 | 0.489 | 0.489 | 0.483 | 0.404 | 0.496 | 0.390 | 0.347 | 0.408 | 0.169 | 0.232 | 0.401 | 0.000 |  |  |  |  |  |  |  |  |  |  |  |  |  |  |  |  |  |  |  |  |
| WXZ | 0.672 | 0.576 | 0.498 | 0.568 | 0.586 | 0.544 | 0.581 | 0.613 | 0.555 | 0.520 | 0.524 | 0.515 | 0.449 | 0.532 | 0.479 | 0.416 | 0.519 | 0.273 | 0.352 | 0.535 | 0.399 | 0.000 |  |  |  |  |  |  |  |  |  |  |  |  |  |  |  |  |  |  |  |
| LDZ | 0.620 | 0.532 | 0.487 | 0.509 | 0.531 | 0.511 | 0.522 | 0.588 | 0.566 | 0.531 | 0.491 | 0.500 | 0.419 | 0.507 | 0.414 | 0.386 | 0.471 | 0.232 | 0.276 | 0.403 | 0.173 | 0.415 | 0.000 |  |  |  |  |  |  |  |  |  |  |  |  |  |  |  |  |  |  |
| LBZ | 0.637 | 0.533 | 0.493 | 0.518 | 0.533 | 0.512 | 0.541 | 0.579 | 0.569 | 0.527 | 0.515 | 0.509 | 0.408 | 0.493 | 0.411 | 0.376 | 0.431 | 0.235 | 0.223 | 0.405 | 0.175 | 0.401 | 0.187 | 0.000 |  |  |  |  |  |  |  |  |  |  |  |  |  |  |  |  |  |
| MPZ | 0.633 | 0.512 | 0.454 | 0.464 | 0.502 | 0.466 | 0.499 | 0.544 | 0.531 | 0.473 | 0.484 | 0.476 | 0.369 | 0.444 | 0.357 | 0.364 | 0.522 | 0.298 | 0.359 | 0.472 | 0.252 | 0.416 | 0.251 | 0.296 | 0.000 |  |  |  |  |  |  |  |  |  |  |  |  |  |  |  |  |
| GYZ | 0.645 | 0.548 | 0.473 | 0.508 | 0.535 | 0.517 | 0.552 | 0.632 | 0.570 | 0.526 | 0.536 | 0.517 | 0.419 | 0.519 | 0.391 | 0.359 | 0.483 | 0.204 | 0.247 | 0.500 | 0.172 | 0.429 | 0.262 | 0.146 | 0.392 | 0.000 |  |  |  |  |  |  |  |  |  |  |  |  |  |  |  |
| XJZ | 0.638 | 0.546 | 0.482 | 0.510 | 0.531 | 0.511 | 0.549 | 0.624 | 0.576 | 0.513 | 0.544 | 0.527 | 0.421 | 0.524 | 0.402 | 0.375 | 0.506 | 0.239 | 0.282 | 0.500 | 0.203 | 0.451 | 0.264 | 0.126 | 0.375 | 0.069 | 0.000 |  |  |  |  |  |  |  |  |  |  |  |  |  |  |
| ZXZ | 0.605 | 0.493 | 0.452 | 0.485 | 0.514 | 0.486 | 0.483 | 0.534 | 0.516 | 0.504 | 0.498 | 0.489 | 0.404 | 0.498 | 0.363 | 0.368 | 0.484 | 0.254 | 0.269 | 0.430 | 0.211 | 0.409 | 0.242 | 0.223 | 0.241 | 0.316 | 0.279 | 0.000 |  |  |  |  |  |  |  |  |  |  |  |  |  |
| DSZ | 0.597 | 0.492 | 0.440 | 0.447 | 0.479 | 0.431 | 0.452 | 0.477 | 0.478 | 0.439 | 0.443 | 0.445 | 0.325 | 0.421 | 0.274 | 0.290 | 0.483 | 0.139 | 0.225 | 0.418 | 0.234 | 0.360 | 0.237 | 0.284 | 0.231 | 0.345 | 0.331 | 0.175 | 0.000 |  |  |  |  |  |  |  |  |  |  |  |  |
| HSZ | 0.672 | 0.599 | 0.542 | 0.563 | 0.578 | 0.543 | 0.561 | 0.640 | 0.616 | 0.544 | 0.558 | 0.557 | 0.479 | 0.545 | 0.492 | 0.487 | 0.565 | 0.309 | 0.400 | 0.599 | 0.417 | 0.520 | 0.369 | 0.435 | 0.417 | 0.546 | 0.500 | 0.370 | 0.229 | 0.000 |  |  |  |  |  |  |  |  |  |  |  |
| YAZ | 0.623 | 0.519 | 0.442 | 0.460 | 0.501 | 0.438 | 0.442 | 0.547 | 0.498 | 0.436 | 0.474 | 0.444 | 0.347 | 0.443 | 0.312 | 0.311 | 0.518 | 0.161 | 0.270 | 0.524 | 0.221 | 0.461 | 0.307 | 0.323 | 0.291 | 0.381 | 0.391 | 0.254 | 0.094 | 0.316 | 0.000 |  |  |  |  |  |  |  |  |  |  |
| CBZ | 0.680 | 0.625 | 0.551 | 0.568 | 0.578 | 0.554 | 0.602 | 0.671 | 0.633 | 0.555 | 0.575 | 0.583 | 0.503 | 0.566 | 0.550 | 0.523 | 0.598 | 0.351 | 0.427 | 0.645 | 0.422 | 0.567 | 0.427 | 0.420 | 0.490 | 0.533 | 0.510 | 0.438 | 0.341 | 0.247 | 0.391 | 0.000 |  |  |  |  |  |  |  |  |  |
| GQZ | 0.628 | 0.534 | 0.484 | 0.524 | 0.552 | 0.514 | 0.486 | 0.537 | 0.527 | 0.472 | 0.496 | 0.496 | 0.401 | 0.498 | 0.376 | 0.383 | 0.525 | 0.248 | 0.317 | 0.460 | 0.266 | 0.397 | 0.315 | 0.274 | 0.332 | 0.342 | 0.331 | 0.276 | 0.207 | 0.364 | 0.145 | 0.358 | 0.000 |  |  |  |  |  |  |  |  |
| AFZ | 0.629 | 0.543 | 0.499 | 0.509 | 0.532 | 0.498 | 0.504 | 0.533 | 0.528 | 0.480 | 0.489 | 0.501 | 0.355 | 0.464 | 0.319 | 0.353 | 0.550 | 0.240 | 0.303 | 0.498 | 0.314 | 0.443 | 0.343 | 0.264 | 0.354 | 0.325 | 0.322 | 0.265 | 0.196 | 0.358 | 0.189 | 0.332 | 0.162 |  | 0.000 |  |  |  |  |  |  |
| BMZ | 0.653 | 0.584 | 0.524 | 0.529 | 0.549 | 0.515 | 0.546 | 0.564 | 0.567 | 0.486 | 0.487 | 0.513 | 0.360 | 0.485 | 0.326 | 0.359 | 0.622 | 0.242 | 0.390 | 0.553 | 0.387 | 0.469 | 0.390 | 0.373 | 0.360 | 0.429 | 0.408 | 0.358 | 0.208 | 0.362 | 0.266 | 0.340 | 0.210 |  | 0.145 | 0.000 |  |  |  |  |  |
| LHZ | 0.679 | 0.600 | 0.533 | 0.544 | 0.568 | 0.531 | 0.554 | 0.606 | 0.597 | 0.500 | 0.524 | 0.530 | 0.377 | 0.487 | 0.355 | 0.391 | 0.622 | 0.274 | 0.400 | 0.617 | 0.402 | 0.510 | 0.434 | 0.410 | 0.405 | 0.470 | 0.462 | 0.379 | 0.243 | 0.340 | 0.251 | 0.416 | 0.230 |  | 0.219 | 0.159 | 0.000 |  |  |  |  |
| PMZ | 0.688 | 0.613 | 0.541 | 0.558 | 0.585 | 0.544 | 0.562 | 0.635 | 0.599 | 0.506 | 0.533 | 0.532 | 0.392 | 0.511 | 0.384 | 0.418 | 0.655 | 0.295 | 0.430 | 0.649 | 0.416 | 0.552 | 0.483 | 0.423 | 0.441 | 0.490 | 0.435 | 0.395 | 0.273 | 0.427 | 0.229 | 0.482 | 0.216 |  | 0.208 | 0.147 | 0.076 | 0.000 |  |  |  |
| HGZ | 0.699 | 0.628 | 0.562 | 0.573 | 0.588 | 0.556 | 0.595 | 0.636 | 0.618 | 0.534 | 0.552 | 0.563 | 0.410 | 0.514 | 0.421 | 0.455 | 0.637 | 0.323 | 0.439 | 0.648 | 0.446 | 0.549 | 0.482 | 0.439 | 0.443 | 0.510 | 0.501 | 0.431 | 0.307 | 0.389 | 0.346 | 0.423 | 0.265 |  | 0.248 | 0.145 | 0.030 | 0.135 | 0.000 |  |  |
| MHZ | 0.628 | 0.535 | 0.488 | 0.505 | 0.532 | 0.493 | 0.496 | 0.519 | 0.539 | 0.471 | 0.488 | 0.499 | 0.376 | 0.481 | 0.313 | 0.352 | 0.550 | 0.238 | 0.339 | 0.513 | 0.355 | 0.425 | 0.352 | 0.382 | 0.300 | 0.434 | 0.405 | 0.269 | 0.147 | 0.197 | 0.229 | 0.358 | 0.241 |  | 0.216 | 0.139 | 0.099 | 0.200 | 0.162 | 0.000 |  |
| XSZ | 0.702 | 0.651 | 0.579 | 0.590 | 0.597 | 0.569 | 0.609 | 0.704 | 0.653 | 0.564 | 0.589 | 0.599 | 0.488 | 0.551 | 0.542 | 0.535 | 0.645 | 0.379 | 0.473 | 0.667 | 0.471 | 0.590 | 0.445 | 0.440 | 0.475 | 0.571 | 0.529 | 0.465 | 0.337 | 0.245 | 0.409 | 0.117 | 0.369 |  | 0.327 | 0.305 | 0.410 | 0.449 | 0.403 | 0.366 | 0.000 |
